# Integrative Human Genetic and Cellular Analysis of the Pathophysiological Roles of AnxA2 in Alzheimer’s Disease

**DOI:** 10.3390/antiox13101274

**Published:** 2024-10-21

**Authors:** Lianmeng Ye, Jiazheng Zhao, Zhengpan Xiao, Wenyu Gu, Xiaoxuan Liu, Nuela Manka’a Che Ajuyo, Yi Min, Yechun Pei, Dayong Wang

**Affiliations:** 1 Laboratory of Biopharmaceuticals and Molecular Pharmacology, Key Laboratory of Tropical Biological Resources of the Ministry of Education of China, School of Pharmaceutical Sciences, Hainan University, Haikou 570228, China; 2 One Health Cooperative Innovation Center, Hainan University, Haikou 570228, China; 3 Department of Biotechnology, School of Life and Health Sciences, Hainan University, Haikou 570228, China

**Keywords:** Alzheimer’s disease, annexin A2, human genetic analysis, amyloid β peptide, cellular activity

## Abstract

Alzheimer’s disease (AD) is an intractable and progressive neurodegenerative disease. Amyloid beta (Aβ) aggregation is the hallmark of AD. Aβ induces neurotoxicity through a variety of mechanisms, including interacting with membrane receptors to alter downstream signaling, damaging cellular or organelle membranes, interfering with protein degradation and synthesis, and inducing an excessive immune-inflammatory response, all of which lead to neuronal death and other pathological changes associated with AD. In this study, we extracted gene expression profiles from the GSE5281 and GSE97760 microarray datasets in the GEO (Gene Expression Omnibus) database, as well as from the Human Gene Database. We identified differentially expressed genes in the brain tissues of AD patients and healthy persons. Through GO, KEGG, and ROC analyses, annexin A2 (AnxA2) was identified as a putative target gene. Notably, accumulating evidence suggests that intracellular AnxA2 is a key regulator in various biological processes, including endocytosis, transmembrane transport, neuroinflammation, and apoptosis. Thus, we conducted a series of cell biology experiments to explore the biological function of AnxA2 in AD. The results indicate that AnxA2 gene knockdown primarily affects oxidative phosphorylation, cell cycle, AD, protein processing in the endoplasmic reticulum, SNARE interactions in vesicular transport, and autophagy. In SH-SY5Y cells secreting Aβ42, AnxA2 gene knockdown exacerbated Aβ42-induced cytotoxicity, including cell death, intracellular ROS levels, and neuronal senescence, altered cell cycle, and reduced ATP levels, suggesting its critical role in mitochondrial function maintenance. AnxA2 gene knockdown also exacerbated the inhibitory effect of Aβ42 on cell migration. AnxA2 overexpression reduced the inflammatory response induced by Aβ42, while its absence increased pro-inflammatory and decreased anti-inflammatory responses. Furthermore, AnxA2 gene knockdown facilitated apoptosis and decreased autophagy. These results indicated potential pathophysiological roles of AnxA2 in AD.

## 1. Introduction

Alzheimer’s Disease (AD) is a neurodegenerative disease. AD symptoms begin with mild memory loss and proceed to severe cognitive dysfunction, behavioral abnormalities, and social disorders [1]. Fifty million people are currently living with AD, which is expected to be three times as high by the year 2050 [2]. Many hypotheses about the pathogenesis of AD have been proposed. The fundamental pathophysiology of AD is known to be the deposition of amyloid β peptide (Aβ), hyperphosphorylation of Tau protein, oxidative stress, mitochondrial cascade, inflammatory response, and disruption of the insulin-signaling pathway. However, Alzheimer’s disease is a complex disease that is affected by genetic and environmental factors. Thus, a single hypothesis may not fully explain the overall incidence of AD [3,4]. The presence of insoluble aggregates of Aβ in the form of neurotic plaques (NPs) is one of the main features that define Alzheimer’s disease. Soluble Aβ protein may interact with membrane or intracellular receptors [5,6,7,8] and activate downstream pathways to promote the generation of reactive oxygen species (ROS), hyperphosphorylation of Tau protein, and cause inflammation, ultimately leading to neuronal death and other pathological changes associated with AD.

The inflammatory hypothesis of AD posits that chronic inflammatory responses within the brain are a key factor in the pathogenesis of AD. This hypothesis is supported by multiple findings: the presence of abundant activated microglia and astrocytes in the brains of AD patients [9], along with elevated levels of various pro-inflammatory mediators [10]. The activation of these glial cells is typically accompanied by the release of pro-inflammatory cytokines such as interleukin-1β (IL-1β), tumor necrosis factor-α (TNF-α), and interleukin-6 (IL-6) [11,12]. In AD research, the oxidative stress toxicity of Aβ itself, alterations in mitochondrial function, and metal ion toxicity have been extensively reported [13,14]. Aβ exerts neurotoxicity through mechanisms such as oxidative damage to lipids, proteins, and DNA, as well as by reducing the activity of antioxidant enzymes. Additionally, impaired mitochondrial function may further exacerbate Aβ-induced neurotoxicity and promote the accumulation of cellular ROS. The deposition of transition metals in the AD brain further facilitates Aβ aggregation, thereby accelerating the progression of AD [15]. Consequently, Aβ aggregates may induce excessive ROS production through mitochondrial damage or metal ion homeostasis imbalance. The presence of numerous autophagosomes and autolysosomes, accompanied by autophagy dysfunction, has been observed in the brains of AD patients [16,17]. Increasing evidence suggests that autophagy is crucial for maintaining the structure of complex hippocampal neurons and cortical circuits, and abnormalities in the autophagy pathway are considered a major cause of Aβ-mediated AD pathogenesis [18]. Autophagy may be involved in the AD pathophysiological process by regulating the production and clearance of Aβ. Moreover, Aβ-mediated autophagy impairment may be associated with mitochondrial dysfunction, endoplasmic reticulum stress, and oxidative stress [19,20,21,22].

Autophagy is a highly conserved cellular mechanism wherein aggregation-prone proteins and damaged organelles are targeted for lysosomal degradation [23]. The autophagic process initiates with an isolation membrane, also known as the phagophore, which expands to engulf intracellular cargo such as protein aggregates and organelles, sequestering them within a double-membraned autophagosome [24]. The loaded autophagosome fuses with the lysosome, promoting the degradation of its contents [25]. Organelle membrane fusion is mediated by soluble N-ethylmaleimide-sensitive factor attachment protein receptor (SNARE) complexes. Upon membrane contact, specific SNARE complexes in each organelle interact with their counterparts in the opposing organelle to mediate membrane fusion. For instance, the fusion of the late endosome and lysosome uses Syntaxin 7, Vti1b, and Syntaxin 8 on the late endosome and VAMP7 on the lysosome [26,27,28,29,30]. In the autophagy pathway, studies have shown that VAMP7, VAMP8, and Vti1b play roles in the fusion of autolysosomes in mammals [31,32,33]. Syntaxin 17 (Stx17) and VAMP8 have been reported to mediate autophagosome-lysosome fusion [34]. In addition to providing the core machinery for membrane fusion, SNARE-interacting proteins can facilitate the formation of the complex. For example, SNAP29 and Atg14 stabilize the Stx17-VAMP8 interaction, promoting autophagosome-lysosome fusion [35,36,37].

**Figure 1:**
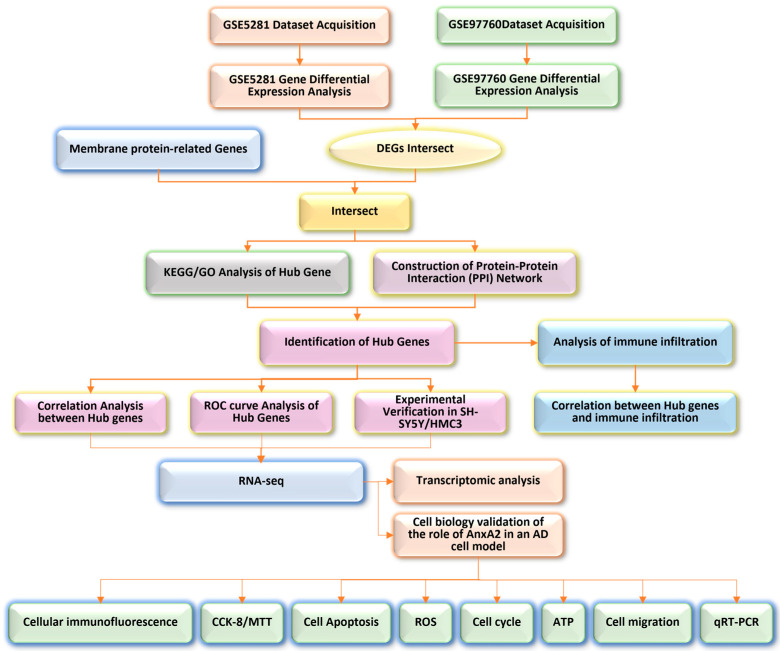
Scheme of experimental design.

In this study, we extracted gene expression profiles from the GSE5281 and GSE97760 microarray datasets in the GEO database, as well as from the Human Gene (HG) Database, to identify gene biomarkers associated with AD. Through detailed analyses, AnxA2 was identified as a putative target gene. Subsequently, transcriptomic analysis and a series of cell biology experiments were conducted to explore the pathophysiological role of AnxA2 in AD (Figure 1). Interestingly, within the annexin family, AnxA1 acts as an anti-inflammatory molecule, participating in Aβ42 clearance by activating neprilysin and regulating the RhoA-ROCK signaling pathway [38], in contrast, AnxA2, in addition to its role as an anti-inflammatory molecule, is also involved in the pro-inflammatory response mediated by tissue plasminogen activator (tPA) [39]. AnxA2 knockout mice exhibit increased levels of proinflammatory cytokines, such as TNF-α, following traumatic brain injury (TBI) due to reduced inflammasome activation [40]. Moreover, AnxA2 may promote the release of inflammatory factors and the migration of inflammatory cells by interacting with lipids and proteins on the cell membrane, thereby participating in the inflammatory response [39]. The absence of AnxA2 exacerbates the pro-inflammatory response through IL-17 signaling [41]. Studies have also shown that AnxA2 plays a crucial regulatory role in oxidative stress processes; it can maintain intracellular redox balance by modulating the generation and clearance of ROS, thereby protecting cells from oxidative stress damage [42]. In AD, Aβ accumulation leads to increased ROS production, triggering oxidative stress, which further exacerbates neuronal injury and death. Additionally, AnxA2 is implicated in autophagy, where it binds to presenilin 1 (PS1) phosphorylated at the Ser367 site, facilitating autophagosome-lysosome fusion and reducing Aβ42 accumulation by increasing the degradation of amyloid precursor protein-β-C-terminal fragment through autophagy [43]. Furthermore, double immunofluorescence staining confirmed elevated AnxA2 expression in astrocytes and neurons [44,45]. Similar to AnxA1, AnxA2 translocates to membrane-associated partners, such as EGFR, and is linked to enhanced disease pathogenicity. In summary, the precise role of AnxA2 in AD remains unclear. Here, we employed molecular and cellular biology techniques to analyze the effects of AnxA2 gene silencing on inflammation, apoptosis, oxidative stress, and autophagy. These findings contribute to a better understanding of the role of annexins and vesicular transport in AD pathophysiology and provide insights into the potential of AnxA2 as a therapeutic target.

## 2. Materials and Methods

### 2.1. Membrane Protein-Related Genes Datasets and Microarray Data

A total of 20,877 genes were obtained from the GeneCard: The Human Gene Database (https://www.genecards.org/, accessed on 26 August 2022). The mRNA expression profile datasets of GSE5281 and GSE97760 were downloaded from GEO (https://www.ncbi.nlm.nih.gov/geo/, accessed on 26 August 2022). GSE5281 is in GPL570 ({HG-U133_Plus_2} Affymetrix Human Genome U133 Plus 2.0 Array), including 87 AD and 74 non-AD control samples. GSE97760 is on GPL16699 platform (Agilent039494-SurePrint G3 Human GE v2 8 × 60K Microarray 039381), including AD (n = 9, age 79.3 ± 12.3 years) and age-matched female healthy controls (n = 10, age 72.1 ± 13.1 years).

### 2.2. Identification of DEGs

The normalized expression matrix of microarray data was downloaded from the GSE5281 and GSE97760 datasets. The repeatability of data in them was verified by principal component analysis (PCA). The probes were annotated in the datasets. Additionally, the Bioconductor linear models for the microarray data package (http://www.bioconductor.org/packages/release/bioc/html/limma.html, accessed on 26 August 2022) were used to identify DEGs by comparing the expression values between AD and the control tissues. An FDR-adjusted *p*-value less than 0.05 and |logFC| > 2 were used as the selection criteria. The Bioconductor heatmaps software (version 3.19) in R language (vesion 4.4) was used to draw the heatmaps of the DEGs.

### 2.3. Overlapping of Alzheimer’s Disease-Related Module Genes with Membrane Protein-Related Genes

Membrane protein-related genes were downloaded from the aforementioned GeneCard. We overlapped these genes with AD-related module genes derived from GSE5281 and GSE97760. The Veen diagram was used to describe the details of the hub genes.

### 2.4. Gene Ontology (GO) and KEGG Pathway Analysis of Hub Genes

GO and KEGG pathway enrichment analyses were conducted using the “GO plot” package in R software (version 4.4). The GO analysis consisted of cellular components (CC), biological processes (BP), and molecular function (MF). Moreover, the clusterProfiler (3.18.0) in Bioconductor was used to perform the GO and KEGG pathway analysis of the DEGs. An FDR-adjusted *p*-value less than 0.05 was considered statistically significant.

### 2.5. Protein-Protein Interaction (PPI) Establishment and Identification of Hub Genes

PPI of Hub genes was analyzed using STRING (Search Tool for the Retrieval of Interacting Genes/Proteins, version 11.5) database (https://cn.string-db.org/, accessed on 26 August 2022) and Cytoscape software (version 3.8.1). The correlation analysis of the differentially expressed autophagy-related genes was identified using Spearman correlation in the “corrplot” package of R software (version 4.4).

### 2.6. Immune Infiltration by CIBERSORT Analysis

The CIBERSORT algorithm was used to analyze the normalized gene expression data obtained previously, and the proportions of 22 kinds of immune cells were obtained. These immune cells included naive B cells, memory B cells, plasma cells, CD8+ T cells, naive CD4+ T cells, resting memory CD4+ T cells, activated memory CD4+ T cells, follicular helper T cells, regulatory T cells (Tregs), gamma delta T cells, resting NK cells, activated NK cells, monocytes, M0, M1 and M2 macrophages, resting dendritic cells, activated dendritic cells, resting mast cells, activated mast cells, eosinophils and neutrophils. A *p*-value less than 0.05 was used to screen the samples, and the percentage of each type of immune cell in the samples was computed. PCA was performed to determine whether there was a difference in immune cell infiltration between the synovial tissue of AD patients and normal controls. Furthermore, the different immune infiltration levels of each immune cell between the two groups were analyzed using the vioplot package (3.6.0) in R language (version 4.4).

### 2.7. Construction and Validation of the Logistic Regression

Logistic regression was constructed to effectively differentiate AD patients from controls. To evaluate the performance of the logistic regression model for predicting the occurrence of AD, we performed receiver operating characteristic (ROC) curve analyses using the pROC package (version 1.18.5) for R software (version 4.4) [46]. Furthermore, we selected the statistically significant genes from hub genes (*p* < 0.05) and used the nomogram to predict the occurrence of AD. The violin plot showed the expression levels of the hub genes.

### 2.8. RNA Extraction and Quantitative Real-Time Polymerase Chain Reaction (qRT-PCR) Validation of the Hub Genes

The Aβ-secreting and non-secreting SH-SY5Y or HMC3 cells were harvested for qRT-PCR validation. Total RNA was extracted from cells with an RNA Extraction Kit (Omega, Guangzhou, China). Reverse transcription was conducted using the PrimeScript RT Master Mix Kit (Takara, Dalian, China). The mRNA level was assessed using TB Green Premix Ex Taq Kit (Takara, Dalian, China). Transcription levels of mRNA were calculated by the 2^−ΔΔCt^ method with the normalization to β-actin. Moreover, one-way analysis of variance (ANOVA) was used for the statistical analysis, and *p* < 0.05 indicated a significant difference.

### 2.9. Cell Culture

SH-SY5Y and HMC3 cells taken out from the liquid nitrogen were rapidly thawed at 37 °C. After re-suspension in DMEM containing 10% fetal bovine serum and antibiotics, the cells were transferred to cell culture dishes coated with the gelatin made from deep-sea fish skin and then cultured in a CO_2_ incubator at 37 °C. When the cultured cells reached 80% confluency, they were used for experiments.

### 2.10. Transfection and Expression of Secreted Aβ42

The medium was replaced with the Opti-MEM 2 h before transfection. Then, the cells were transfected with pcDNA3.1-Aβ42 plasmids for 5 h, in which the sequence encoding the signal peptide for secretion was added to the N terminus of Aβ42. Five hours after transfection, it was switched to a DMEM medium containing 10% FBS, penicillin, and streptomycin. 24 h after transfection, the antibiotic G418 was added to the culture.

### 2.11. siRNA Transfection and ELISA Detection

A similar aforementioned transfection procedure was adopted. The synthesized siRNA (Sangon, Shanghai, China) was diluted to 10 μM and combined with 10 μL of Lipofectamine 2000 (Sigma, St. Louis, MO, USA), which had been pre-incubated at room temperature for 15 min, and Opti-MEM was added to the culture plates. The plates were then incubated at 37 °C for 4 h. After transfection, the medium was replaced with DMEM (ThermoFisher, Waltham, MA, USA) containing 5% fetal bovine serum (16010159, Biological Industries Ltd., Heamek, Israel) and antibiotics (penicillin and streptomycin). 24 h after transfection, the antibiotic G418 was added to the culture. After the positive selection, 100 µL of the culture medium was transferred to a 96-well high-binding plate and incubated overnight at 4 °C. Wash the wells four times with PBS, then block with 5% non-fat dry milk for 2 h. Add the primary antibody (1:200) and incubate overnight at 4 °C. After washing four times with PBS, add the horseradish peroxidase-conjugated secondary antibody (1:1000). Process using an EL-TMB kit (C520026, Sangon Biotech, Shanghai, China) and measure the luminescence by using a microplate reader (AMR-100, Allsheng Instruments Co., Ltd., Hangzhou, China).

### 2.12. MTT Assay

The SH-SY5Y cells were transfected with pcDNA3.1 plasmids expressing secretable Aβ42, AnxA2 alone, AnxA2 together with P11, and siRNA targeting AnxA2 gene, or AnxA2 and P11. After transfection, the cells were transferred into the wells of 96-well plates at the amount of 5 × 10^3^ cells per well. The cells not transfected were used as control groups. 48 h after incubation at 37 °C and 5% CO_2_, the medium was aspirated, and 90 µL of serum-free medium and 10 µL of MTT solution (5 mg/mL) were added to each well. 4 h later, the wells were aspirated, 100 µL of DMSO was added, and the plates were shaken for 10 min at 37 °C. The optical density (OD) of each well was measured at 570 nm in a Synergy H1 microplate reader, and the mean value of the blank control was used as the 100% reference value to calculate the cell viability in each well.

### 2.13. CCK-8 Test

The cells transfected in the aforementioned way were seeded into 96-well plates (4 × 10^3^/well) and cultured at 37 °C for 0, 6, 12, 24, 36 and 48 h. Then, the cells were further incubated with 10 µL of CCK-8 solution provided in the kits at 37 °C for an additional 3 h. Then, cell viability was tested by measuring the OD value at the wavelength of 450 nm.

### 2.14. Flow Cytometric Analysis on Reactive Oxygen Species and Apoptosis of Cells Secreting Aβ42

The cells secreting Aβ42 and overexpressing or knocking down AnxA2/P11 genes were adopted. After washing, the cells were stained using PI and the Annexin-V kit (UElandy Inc., Suzhou, China), and cell apoptosis was measured by flow cytometry (CytoFLEX LX, Beckman Coulter Life Sciences, Indianapolis, IN, USA). The data were analyzed using FlowJoTM (BD Biosciences, San Jose, CA, USA). Statistical results from six independent experiments were expressed as the mean ± SEM.

### 2.15. Cell Cycle Assay

Distributions of cell cycles were measured using a Cell Cycle Analysis kit. Cells (1 × 10^5^ cell/mL, 2 mL) were seeded into six-well plates. After treatment, the cells were fixed with ice-cold 70% ethanol at 4 °C overnight. Then, the cells were incubated at 37 °C for 30 min with propidium working solution containing 10 μL of RNase A. Samples were analyzed using a CytoFlex flow cytometer (Beckman Coulter, Brea, CA, USA) at excitation/emission wavelengths of 488/575 nm, and 5000 cells were collected per sample. Data were analyzed using the FlowJo software (version 10.10).

### 2.16. Cellular ATP Assay

The cell culture medium was aspirated, and 200 μL of lysis buffer was added. Then, the cells were fully lysed by trituration. The lysates were collected and centrifuged at 12,000× *g* for 5 min at 4 °C. ATP concentration in supernatants was tested using an ATP detection kit (Beyotime, Shanghai, China): Add 100 μL of ATP detection working solution to each well and incubated at room temperature for 5 min. Then, add 20 μL of the sample or ATP standard and mix well. Immediately measure the corresponding chemiluminescence value by a microplate reader (AMR-100, Allsheng Instruments Co., Ltd., Hangzhou, China) and calculate the ATP concentration in the samples.

### 2.17. Cell Migration Assay

After treatment, cells (1 × 10^5^ cell/mL, 2 mL) were seeded into six-well plates and cultured at 37 °C and 5% CO_2_ 24 h. Uniform scratches were made with pipette tip ends perpendicular to the horizontal lines behind the petri dish, the cells scratched off were washed out with PBS 3 times, and serum-free medium was added. After incubation for 0 h, 6 h, 12 h, and 24 h, the cell images were captured under an optical microscope. The area was analyzed using the ImageJ software (version 1.54). Statistical results from six independent experiments were expressed as the mean ± SEM.

### 2.18. Statistical Analysis

The statistical analyses were performed using the R software (version 3.6.2) or GraphPad Prism 9.5.1 software. Cellular experimental results were analyzed by one- or two-way ANOVA, followed by the Tukey–Kramer test for multiple comparisons. A *p*-value less than 0.05 was considered statistically significant.

## 3. Results

### 3.1. Mining and Validation of Potential Pathogenic Genes for AD from the GEO Database

We compared the AD brain tissue expression datasets (GEO accessions GSE97760 and GSE5281) from GEO with the membrane protein-related genes from the GeneCard: The Human Gene Database in the present study. After batch correction and standardization of the microarray results, DEGs were identified. There were 8180 DEGs in GSE97760 and 9423 in GSE5281 compared to AD patients and healthy controls. Venn analysis indicated 3008 DEGs between the two microarray results (Figure S1A) and 85 overlapped genes between GEO and membrane protein-related genes (Figure S1B). KEGG pathway analysis revealed that the target genes were mainly enriched in the hepatitis B, lipid and atherosclerosis, age-range signaling pathway in diabetic complications, human papillomavirus infection, and small cell lung cancer (Figure S1C). Gene Ontology (GO) functional enrichment analysis showed that the target genes were mainly involved in protein import, regulation of apoptotic signaling pathway, aging, neuron death, and cellular response to peptides (Figure S1D).

The PPI network of the DEGs was analyzed by using STRING (STRING: functional protein association networks (string-db.org)) (Figure S2A). The most significant module, including 20 hub genes, was obtained by using the MCODE plugin of Cytoscape 3.9.1 (Figure S2B). To explore the expression correlation of these target genes, a correlation analysis was performed. The results showed the relationship of the 85 target genes in the GSE5281 and GSE97760 datasets (Figure S3). The landscape of immune infiltration in AD has not been entirely revealed due to technical limitations, especially in subpopulations with a low abundance of cells [47]. Using the CIBERSORT algorithm, the difference in immune infiltration in 22 subpopulations of immune cells between AD and normal tissues was investigated. Figure S4A summarizes the results obtained from 10 normal controls and 9 AD patients. Additionally, a correlation analysis was performed to explore the expression correlation of these immune cells. The results showed the relationship of the 22 subpopulations of immune cells in the GSE97760 dataset (Figure S4B). Compared with normal tissue, AD tissue generally contained a higher proportion of CD8+ T cells (cytotoxic lymphocytes), resting memory CD4+ T cells, and dendritic cells. In contrast, the proportions of regulatory T cells (Tregs), M0 macrophages, and neutrophils were relatively low (Figure S4C, *p* < 0.05).

To verify the role of target genes in AD, 9 genes (MAPK8, NOTCH1, TLR4, AnxA2, APP, CREB1, HIF1A, ITGB1, and KRAS) were selected, GSE5281 and GSE97760 (Figure S5) datasets were brought into our analysis and validation system. The number of AD samples in GSE5281 and GSE97760 was 87 and 9, respectively, and the number of control samples was 74 and 10, respectively. Across the 2 datasets, a significant increase in transcript abundance of these genes was observed in the AD patients compared with that in normal control. The sensitivity and specificity are reflected by the area under the curves (AUC) of the ROC curve. Notably, the results showed that the AUC for these genes in all plots was equal or close to 1, indicating the diagnostic character of these genes. Subsequently, qRT-PCR experiments were conducted to validate the hub genes. The results showed that the relative expression levels of hub genes, including MAPK8, NOTCH1, TLR4, AnxA2, APP, CREB1, HIF1A, ITGB1, and KRAS were consistent with the microarray hybridization. The expression levels of NOTCH1, TLR4, APP, CREB1, HIF1A, ITGB1, and KRAS were significantly higher in Aβ-secreting SH-SY5Y (Figure S6A) or HMC3 (Figure S6B) cells than in non-secreting. In addition, the expression levels of MAPK8 and AnxA2 were significantly decreased.

### 3.2. Transcriptomic Analysis of the Role of AnxA2 in an AD Cell Model Secreting Aβ42

**Figure 2:**
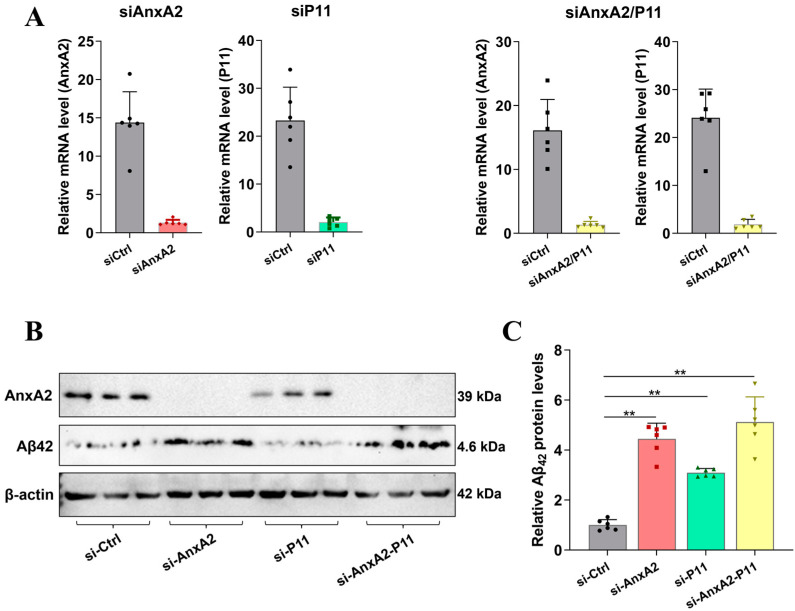
Establishment and validation of AnxA2 gene-knockdown SH-SY5Y cells. (**A**) qRT-PCR validation of AnxA2 and P11 mRNA expression in SH-SY5Y cells treated with siAnxA2; (**B**) Western blotting analysis of AnxA2 and Aβ42 protein expression; (**C**) Semi-quantitation of the expression of the proteins. Data are presented as mean ± SEM. ** *p* < 0.01, determined by one-way ANOVA followed by Tukey–Kramer test for multiple comparisons, n = 6.

**Figure 3:**
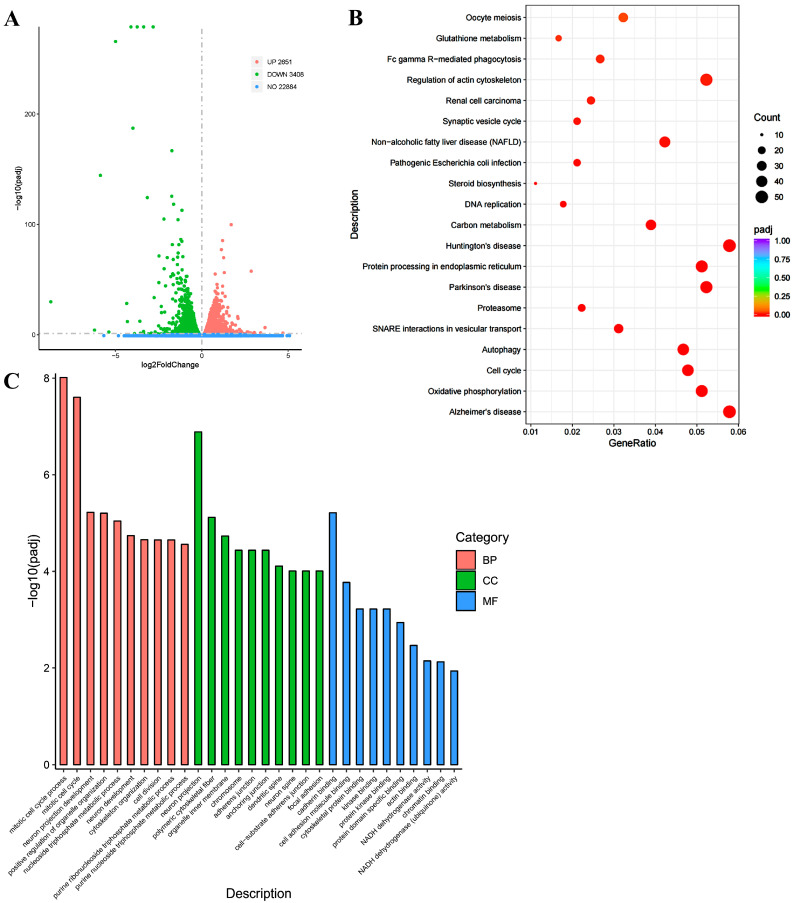
Transcriptomic analysis of differential expression of genes in siAnxA2 treated and non-treated SY-SY5Y cells that secret Aβ42. (**A**) Volcano map of DEGs. The green dots represent upregulated gene transcription, the red dots indicate downregulated transcription, and the blue dots represent nonsignificant transcription. (**B**) KEGG enrichment analysis. (**C**) GO enrichment analysis in the biological process (BP), cellular component (CC), and Molecular function (MF) domains.

To investigate the impact of AnxA2 gene knockdown on the transcriptional level of an AD cell model, AnxA2 siRNA was applied to the cells secreting Aβ42 (Figure 2), followed by Western blotting and transcriptome sequencing. Interestingly, after the AnxA2 gene knockdown (Figure 2A), the protein expression levels of Aβ42 significantly increased (Figure 2B,C). Transcriptomic results showed that the DEGs in the siAnxA2-Aβ42 versus Aβ42 were mainly enriched in the oxidative phosphorylation, cell cycle, AD, protein processing in the endoplasmic reticulum, SNARE interactions in vesicular transport, and autophagy pathways (Figure 3). In the oxidative phosphorylation pathway, there were 20 DEGs, including 12 upregulated and 8 downregulated genes. Among these, the downregulation of mtDNA-encoded complex I subunits DN5 and DN6 is notable, as it is thought to potentially lead to adult optic neuropathies. In the cell cycle pathway, 42 DEGs were identified, including 8 downregulated genes. The expressions of TGF-β (transforming growth factor-β), Ink4c (cyclin-dependent kinase inhibitor 2C), CDK4/6 (cyclin-dependent kinases 4/6), E2F1 (cell cycle transcription factor), and Mad2 (mitotic arrest deficient 2) were decreased. In the AD pathway, the expressions of PSEN and ApoE genes were upregulated, which are prominent in AD pathology. The elevation of PSEN gene expression leads to more production of Aβ42, while APOE4 is associated with increased β-amyloid deposition, tau protein hyperphosphorylation and aggregation, accelerated cognitive decline, and exacerbation of AD pathology. In the SNARE and autophagy pathways, the expressions of the SNARE complex and Vamp8, Stx-17, and Snap29 genes were downregulated. Notably, Stx-17, a member of the Qa-SNARE family, has been shown to be involved in the fusion of autophagosomes with lysosomes through its interaction with Vamp8. In summary, the silence of the AnxA2 gene in the AD cell model affects the level of oxidative phosphorylation, alters the cell cycle, exacerbates cell death, and interferes with the SNARE family genes in the autophagy pathway, affecting autophagosome-lysosome fusion and aggravating AD pathology.

### 3.3. AnxA2 Gene Knockdown Exacerbates Neural Cell Damage Induced by Secreted Aβ42

**Figure 4:**
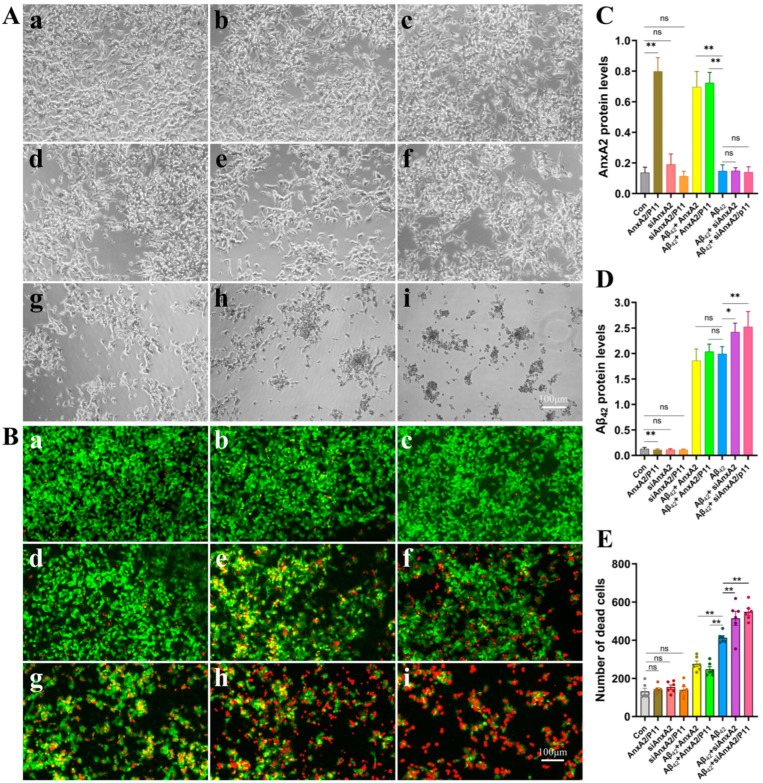
Protective effects of siAnxA2/P11 on SH-SY5Y cells. (**A**) The morphology of SH-SY5Y cells secreting Aβ42. Round-shaped cells with a bright edge are dying or dead. (**B**) Damaged cells were detected using ethidium bromide, a nucleic acid tracer that cannot pass through an intact cell membrane. (**C**) Expression levels of AnxA2 protein in different cell groups. (**D**) Expression levels of Aβ42 protein in different cell groups. (**E**) Quantification of cell damage using the Image J software (version 1.54). Results are expressed as means ± SEM. * *p* < 0.05, ** *p* < 0.01, ns, not significant, analyzed by one-way ANOVA, followed by the Tukey–Kramer test for multiple comparisons, n = 6. In (**A**,**B**): (**a**) Growth of SH-SY5Y cells treated with control medium (Con). (**b**) Growth of SH-SY5Y cells overexpressing AnxA2/P11. (**c**) Growth of SH-SY5Y cells treated with siAnxA2. (**d**) Growth of SH-SY5Y cells treated with siAnxA2/P11. (**e**) Growth of Aβ42-secreting and AnxA2-overexpressing SH-SY5Y cells. (**f**) Growth of Aβ42-secreting and AnxA2/P11-overexpressing SH-SY5Y cells. (**g**) Growth of SH-SY5Y cells secreting Aβ42. (**h**) Growth of Aβ42-secreting SH-SY5Y cells treated with siAnxA2. (**i**) Growth of Aβ42-secreting SH-SY5Y cells treated with siAnxA2/P11.

The aggregation of the Aβ42 protein induces cellular toxicity and damage in vivo, which is a major cause of AD onset. To investigate the impact of AnxA2 on neural cell damage induced by secreted Aβ42, AnxA2 gene knockdown and/or overexpression were performed in the cells secreting Aβ42. Figure 4A presents the morphological changes of the cells in each group. Figure 4B shows the cell death observed under a microscope. Figure 4C,D show the expression levels of AnxA2 and Aβ42 proteins in different groups of cells. Acridine orange (AO, chemical formula C17H19N3) can penetrate intact cell membranes and incorporate into nuclear DNA, emitting bright green fluorescence. Ethidium bromide (EB, chemical formula C21H20BrN3) can only pass through damaged cell membranes, bind to nuclear DNA, and emit orange-red fluorescence. The results show that there is no significant difference in the number of dead cells between the control (Con) group, AnxA2/P11 group, siAnxA2 group, and siAnxA2/P11 group. In the Aβ42 + AnxA2 group and Aβ42 + AnxA2/P11 group, the number of dead cells was significantly reduced by 41.81% and 49.06%, respectively, compared to the Aβ42 group. In contrast, the number of dead cells in the Aβ42 + siAnxA2 group and Aβ42 + siAnxA2/P11 group significantly increased by 86.48% and 94.99%, respectively, compared to the Aβ42 group (Figure 4E). In summary, the AnxA2 gene plays a crucial role in neural cell damage induced by secreted Aβ42 expression. Overexpression of AnxA2 can alleviate neural cell damage caused by Aβ42 while silencing of the AnxA2 gene exacerbates Aβ42-induced neural cell death.

### 3.4. AnxA2 Gene Deletion Exacerbates Aβ42-Induced Cytotoxicity in SH-SY5Y Cells

**Figure 5:**
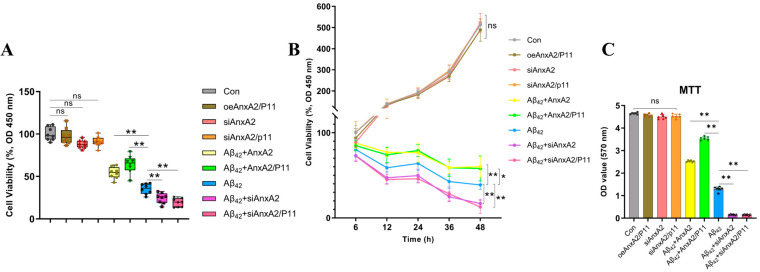
Effects of the AnxA2 on Aβ42-induced cytotoxicity in SH-SY5Y cells. (**A**) CCK-8 assay to detect the effects of AnxA2 on the cytotoxicity induced by secreted Aβ42; (**B**) CCK-8 assay to detect the time-dependent effects of AnxA2 on the cytotoxicity induced by secreted Aβ42; (**C**) MTT assay to detect the effects of AnxA2 on the cytotoxicity induced by secreted Aβ42. Results are expressed as means ± SEM. * *p* < 0.05, ** *p* < 0.01, ns, not significant, analyzed by one-way or two-way ANOVA, followed by the Tukey–Kramer test for multiple comparisons, n = 6.

The aggregation of Aβ42 induces cytotoxicity, causing cellular damage. To investigate this, the cell viability of SH-SY5Y cells secreting Aβ42 under different treatment conditions was assessed by CCK-8 and MTT assays. The viability of cells after 24 h of incubation at 37 °C with 5% CO_2_ is shown in Figure 5; normal cultured cells served as the control group. There was no significant difference in cell viability between the AnxA2/P11 group, siAnxA2 group, siAnxA2/P11 group, and the control group. The cell viability in the Aβ42 group was only 35.34%. The Aβ42 + AnxA2 group and Aβ42 + AnxA2/P11 group showed an increase in cell viability by 18.84% and 28.84%, respectively, compared to the Aβ42 group, whereas the cell viability decreased by 11.04% and 16.08% after AnxA2/P11 gene knockdown (Figure 5A). This indicates that the AnxA2/P11 genes effectively protect cells, and its knockdown increases the cytotoxic effects of Aβ42. Furthermore, Figure 5B demonstrates that the increase in cytotoxicity induced by secreted Aβ42 following AnxA2/P11 gene knockdown is time-dependent. The MTT results also showed that there was no significant difference in cell viability between the Con group, AnxA2/P11 group, siAnxA2 group, and siAnxA2/P11 group. However, cell viability was significantly increased in the Aβ42 + AnxA2 group and Aβ42 + AnxA2/P11 group compared to the Aβ42 group, whereas the cell viability significantly decreased following AnxA2/P11 gene knockdown (Figure 5C).

### 3.5. AnxA2 Gene Silencing Exacerbated Aβ42-Induced Apoptosis

**Figure 6:**
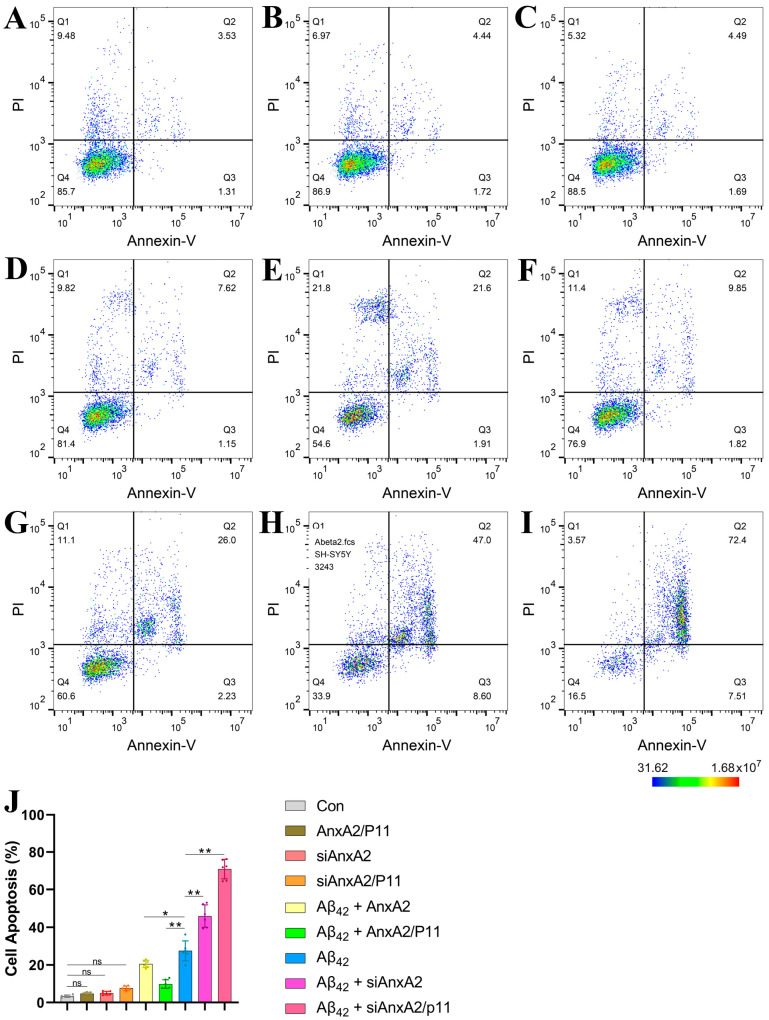
Flow cytometry analysis of the effects of siAnxA2/P11 on cell apoptosis. (**A**) Apoptosis in SH-SY5Y cells treated with control medium (Con). (**B**) Apoptosis in SH-SY5Y cells overexpressing AnxA2/P11. (**C**) Apoptosis in siAnxA2-treated SH-SY5Y cells. (**D**) Apoptosis in siAnxA2/P11-treated SH-SY5Y cells. (**E**) Apoptosis in Aβ42-secreting SH-SY5Y cells overexpressing AnxA2. (**F**) Apoptosis in Aβ42-secreting SH-SY5Y cells overexpressing AnxA2/P11. (**G**) Apoptosis in SH-SY5Y cells secreting Aβ42. (**H**) Apoptosis in Aβ42-secreting SH-SY5Y cells treated with siAnxA2. (**I**) Apoptosis in Aβ42-secreting SH-SY5Y cells treated with siAnxA2/P11. (**J**) Quantification of apoptosis in the SH-SY5Y cells using FlowJo (version 10.10). In figure (**A**) to (**I**), the color represents cell density. In figure (**J**), the results are expressed as mean ± SEM. * *p* < 0.05, ** *p* < 0.01, ns, not significant, analyzed by one-way ANOVA, followed by the Tukey–Kramer test for multiple comparisons, n = 6.

Neurodegeneration in AD is complex, involving various types of neuronal cell death that play crucial roles individually or in combination. Different forms of cell death, including apoptosis, necroptosis, pyroptosis, and ferroptosis, are not only involved in clearing damaged or obsolete cells but also play important roles in inhibiting pathogen spread. Observations under optical (Figure 4A) and fluorescence microscopes (Figure 4B) indicate that the AnxA2 gene plays a key role in neuronal cell death induced by secreted Aβ42. Therefore, we used flow cytometry to analyze the impact of AnxA2 on apoptosis in SH-SY5Y and HMC3 cells induced by secreted Aβ42. The experimental results showed no significant difference in apoptosis levels among the Con group (Figure 6A), AnxA2/P11 group (Figure 6B), siAnxA2 group (Figure 6C), and siAnxA2/P11 group (Figure 6D). However, the apoptosis levels in the Aβ42 + AnxA2 group (Figure 6E) and Aβ42 + AnxA2/P11 group (Figure 6F) were significantly reduced by 16.92% and 33.15%, respectively, compared to the Aβ42 group (Figure 6G). In contrast, the apoptosis levels in the Aβ42 + siAnxA2 group (Figure 6H) and Aβ42 + siAnxA2/P11 group (Figure 6I) were significantly increased by 103.54% and 213.92%, respectively, compared to the Aβ42 group (Figure 6J). Similar experiments conducted on HMC3 cells yielded comparable results (Figure S7). In summary, Aβ42 induces neuronal apoptosis, and the silencing of the AnxA2 gene exacerbates the apoptotic process.

### 3.6. AnxA2 Gene Knockdown Exacerbates Aβ42-Increased Reactive Oxygen Species Levels

**Figure 7:**
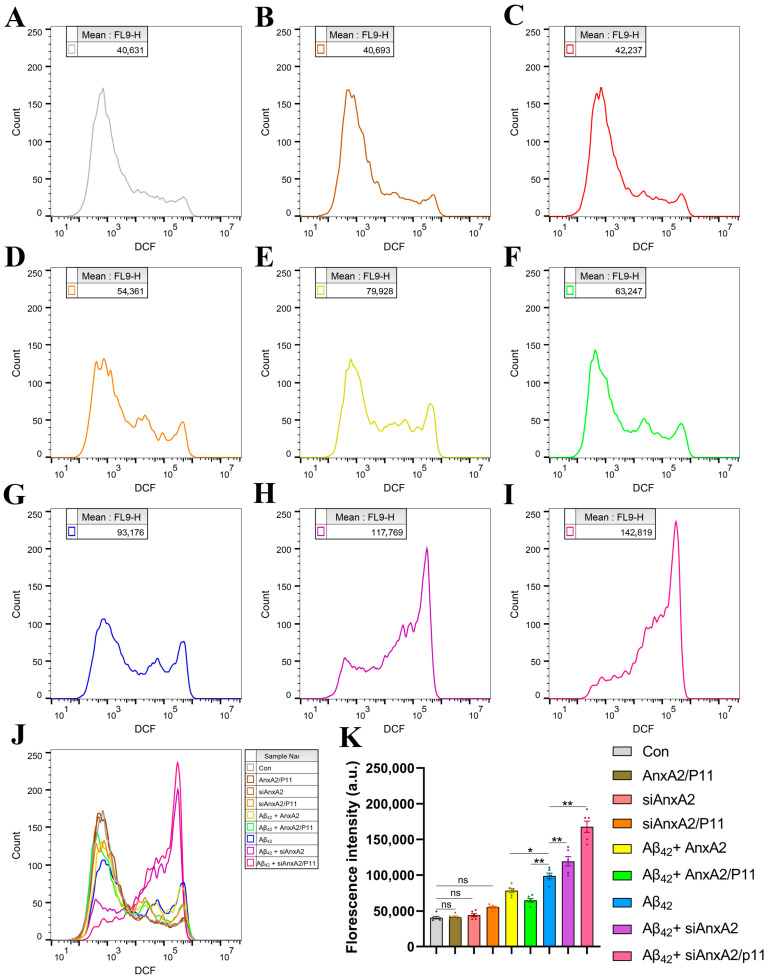
Effects of siAnxA2/P11 on ROS levels in SH-SY5Y cells secreting Aβ42. (**A**) ROS in wild-type SH-SY5Y cells (control). (**B**) ROS produced in SH-SY5Y cells overexpressing AnxA2/P11. (**C**) ROS levels in siAnxA2-treated SH-SY5Y cells. (**D**) ROS levels in siAnxA2/P11-treated SH-SY5Y cells. (**E**) ROS levels in Aβ42-secreting SH-SY5Y cells overexpressing AnxA2. (**F**) ROS levels in Aβ42-secreting SH-SY5Y cells overexpressing AnxA2/P11. (**G**) ROS levels in Aβ42-secreting SH-SY5Y cells. (**H**) ROS levels in Aβ42-secreting SH-SY5Y cells treated with siAnxA2. (**I**) ROS levels in Aβ42-secreting SH-SY5Y cells treated with siAnxA2/P11. (**J**) The overlay of flow cytometry charts is shown in the figure (**A**–**I**). (**K**) Quantification of the ROS levels using FlowJoTM. Results are expressed as mean ± SEM. * *p* < 0.05, ** *p* < 0.01, ns, not significant, analyzed by one-way ANOVA, followed by the Tukey–Kramer test for multiple comparisons, n = 6.

Reactive oxygen species (ROS) may be byproducts of physiological metabolism, with multiple sources within the body. Abnormal levels of ROS disrupt the antioxidant system and cause oxidative stress. Increasing evidence suggests that oxidative stress may be one of the key factors in cognitive aging and the onset of AD. The results of this study show that ROS levels in SH-SY5Y cells secreting Aβ42 are significantly higher compared to the control group (Figure 7G). Overexpression (Figure 7B) or knockdown (Figure 7C,D) of the AnxA2/P11 gene did not alter ROS levels in SH-SY5Y cells. Compared to the Aβ42-secreting group, overexpression of the AnxA2 or AnxA2/P11 gene reduced ROS levels by 20.58% and 34.24%, respectively, whereas knockdown of the AnxA2 or AnxA2/P11 gene increased ROS levels by 20.72% and 69.75% respectively. Additionally, similar experiments conducted on HMC3 cells yielded comparable results (Figure S8). In summary, Aβ42 increases ROS levels and induces oxidative stress in neural cells, and the knockdown of the AnxA2 gene exacerbates ROS levels in the neuronal cell model of AD.

### 3.7. AnxA2 Involvement in the Regulation of the Cell Cycle in SH-SY5Y Cells Secreting Aβ42

**Figure 8:**
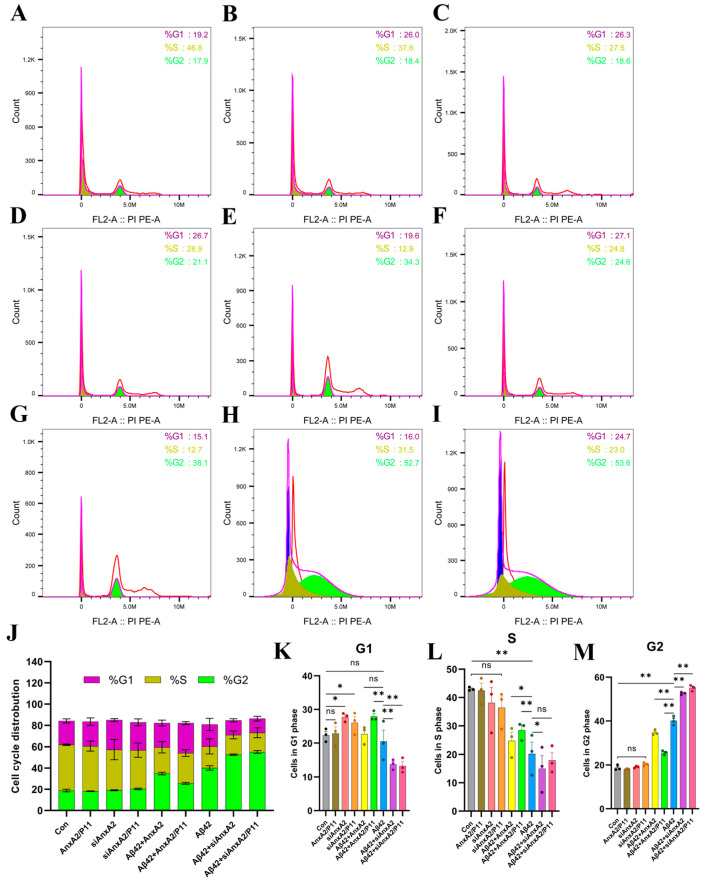
Effects of AnxA2 on cell cycle of SH-SY5Y cells secreting Aβ42. (**A**) Cell cycle of untreated SH-SY5Y cells (Con); (**B**) Cell cycle of SH-SY5Y cells overexpressingAnxA2/P11; (**C**) Cell cycle of siAnxA2-treated SH-SY5Y cells; (**D**) Cell cycle of siAnxA2/P11-treated SH-SY5Y cells; (**E**) Cell cycle of Aβ42-secreting SH-SY5Y cells overexpressing AnxA2; (**F**) Cell cycle of Aβ42-secreting SH-SY5Y cells overexpressing AnxA2/P11; (**G**) Cell cycle of non-treated Aβ42-secreting SH-SY5Y cells; (**H**) Cell cycle of Aβ42-secreting SH-SY5Y cells treated with siAnxA2; (**I**) Cell cycle of Aβ42-secreting SH-SY5Y cells treated with siAnxA2/P11; (**J**) Quantitation of the distribution of different phases of cell cycle including G1 (**K**), S (**L**), and G2 (**M**) phases. Data are presented as mean ± SEM. * *p* < 0.05, ** *p* < 0.01, ns, not significant, determined by one-way ANOVA followed by Tukey–Kramer test for multiple comparisons test, n = 3.

Abnormalities in the cell cycle are closely associated with the development and progress of various diseases. In AD patients, neuronal cell cycle abnormalities are linked to accelerated aging. Post-mitotic neurons that re-enter the cell cycle in the brain age rapidly [48]. There is a close relationship between the cell cycle and apoptosis. The cell cycle is divided into G1 (Gap1), S (DNA synthesis), G2 (Gap2), and M (mitosis). Apoptosis mainly occurs in the G1 and G2 phases and less frequently in the S and M phases [49]. Previous studies have shown that the AnxA2 gene affects apoptosis induced by Aβ42 secretion. In the experiment, we explored whether AnxA2 influences the cell cycle. As shown in Figure 8K, compared to the Con group, overexpression of AnxA2/P11 did not change the proportion of G1 phase cells, while knockdown of AnxA2/P11 significantly increased the proportion of cells in the G1 phase. Compared to the Aβ42 group, overexpression of AnxA2/P11 significantly increases the G1 phase cell proportion, whereas knockdown of AnxA2/P11 significantly reduces it. Figure 8L shows that there was no significant difference in the proportion of S phase cells between the AnxA2/P11 overexpression or knockdown groups and the Con group. However, compared to the Aβ42 group, overexpression of AnxA2/P11 significantly increases the S phase cell proportion, while knockdown of AnxA2 significantly reduces it. Figure 8M indicates no significant difference in the proportion of G2 phase cells between the AnxA2/P11 overexpression or knockout groups and the Con group. The Aβ42 group shows a significantly higher G2 phase cell proportion than the Con group. Compared to the Aβ42 group, overexpression of AnxA2/P11 significantly decreased the G2 phase cell proportion, whereas knockdown of AnxA2 significantly increased it. In a word, Aβ42 secretion alters the cell cycle in SH-SY5Y cells, reducing the S phase cell proportion and increasing the G2 phase cell proportion, thereby accelerating neuronal aging. Knockdown of AnxA2/P11 exacerbates cell cycle abnormalities caused by Aβ42, accelerating neuronal cell aging and apoptosis.

### 3.8. AnxA2 Gene Silencing Aggravates Mitochondrial Dysfunction Induced by Secreted Aβ42

**Figure 9:**
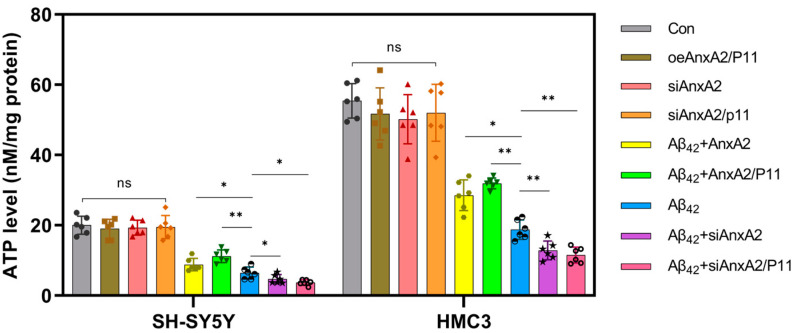
Effects of AnxA2 on intracellular ATP concentration in SH-SY5Y cells and HMC3 cells. Data are presented as mean ± SEM. * *p* < 0.05, ** *p* < 0.01, ns, not significant, determined by one-way ANOVA followed by Tukey–Kramer test for multiple comparisons, n = 6, oeAnxA2/P11: overexpression of AnxA2/P11, si: siRNA.

ATP is the direct energy source within cells and can be used to assess cellular metabolism levels, which has a strong linear relationship with the number of viable cells. In disease states, cholinergic neurons require high energy supplies to maintain cognitive function, and mitochondrial energy failure exacerbates pathophysiological progress and promotes neuronal death. As shown in Figure 9, compared to the Con group, the ATP levels in SH-SY5Y and HMC3 cells did not significantly differ in the AnxA2/P11 gene overexpression and knockdown groups. However, compared to the Con group, ATP levels in the Aβ42-secreting cells were significantly reduced by 67.97% and 66.16% in SH-SY5Y and HMC3 cells, respectively. Compared to the Aβ42 group, ATP levels in the AnxA2 and AnxA2/P11 gene overexpression groups were significantly increased by 37.35%/74.47% and 52.16%/70.22% in SH-SY5Y and HMC3 cells respectively. On the other hand, ATP levels in the Aβ42 + siAnxA2 and Aβ42 + siAnxA2/P11 groups were significantly reduced by 26.99%/42.42% and 31.57%/38.83% in SH-SY5Y and HMC3 cells respectively. These results indicate that, compared to the control group, ATP levels were significantly decreased in the AD cell model, suggesting that Aβ42 accumulation causes mitochondrial dysfunction and AnxA2/P11 gene knockdown exacerbates the pathophysiological process.

### 3.9. AnxA2 Gene Knockdown Aggravated the Inhibitory Effects of Aβ42 on Cell Migration

**Figure 10:**
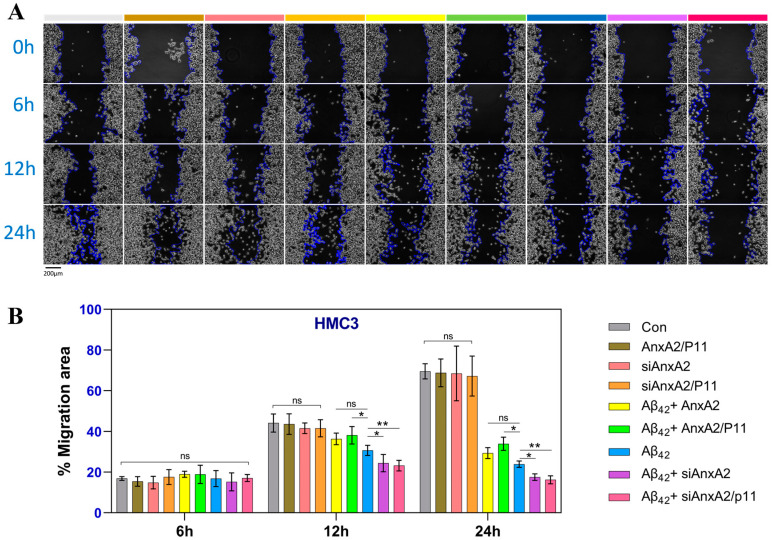
Effects of AnxA2 on HMC3 cell migration. (**A**) Microscopic images of cells at 0 h, 6 h, 12 h, and 24 h after scratching; (**B**) Statistical analysis of cell migration using the ImageJ software. Data are presented as mean ± SEM. * *p* < 0.05, ** *p* < 0.01, ns, not significant, analyzed by one-way ANOVA, followed by Tukey–Kramer test for multiple comparisons, n = 6.

Cell migration, also known as cell crawling, movement, or motility, refers to the movement of cells in response to migration signals or gradients of certain substances. Previous experiments have shown that the secretion of Aβ42 causes cell damage (Figure 4) and apoptosis (Figure 6), and related studies have reported that Aβ42 affects the migration of neural cells [50]. The formation of amyloid plaques and tau protein tangles in the brain are typical pathological features of AD. Due to the migratory capacity of microglia, they aggregate around amyloid plaques and tau protein in AD patients [51]. One of the main processes by which microglia transition from a resting to an activated state is migration and aggregation [52]. To investigate the effects of the AnxA2 on cell migration, a scratch assay was performed. In the experiment shown in Figure 10A, the migration of cells was recorded under a microscope at 0, 6, 12, and 24 h after scratching. The cell migration rate at 6 h was not significantly different among all groups. At 12 h, the Con, AnxA2/P11, siAnxA2, and siAnxA2/P11 groups showed no significant differences, however, compared to the Aβ42-secreting cells, the AnxA2/P11 gene overexpression group showed a significant increase in cell migration rate, while the Aβ42 + siAnxA2 and Aβ42 + siAnxA2/P11 groups showed a significant decrease. At 24 h, the Con, AnxA2/P11, siAnxA2, and siAnxA2/P11 groups did not show significant differences, but the AnxA2/P11 gene-overexpressing group had a significantly increased migration rate compared to the Aβ42-secreting cells, while the Aβ42 + siAnxA2 and Aβ42 + siAnxA2/P11 groups had significantly decreased migration rates. The cell migration rates of the Con, AnxA2/P11, siAnxA2, and siAnxA2/P11 groups increased over time, while the cell migration rates of the Aβ42-secreting cells and AnxA2 gene knockdown cells decreased over time (Figure 10B). Additionally, the same results were obtained in SH-SY5Y cells (Figure S9). In summary, the accumulation of Aβ42 protein inhibits microglia and neural cell migration, and the knockdown of the AnxA2/P11 gene exacerbates the inhibitory effects of Aβ42 on cell migration.

### 3.10. AnxA2 Gene Knockdown Upregulates Pro-Inflammatory Cytokine Transcription and Downregulates Anti-Inflammatory Cytokine Transcription in AD Cell Model

**Figure 11:**
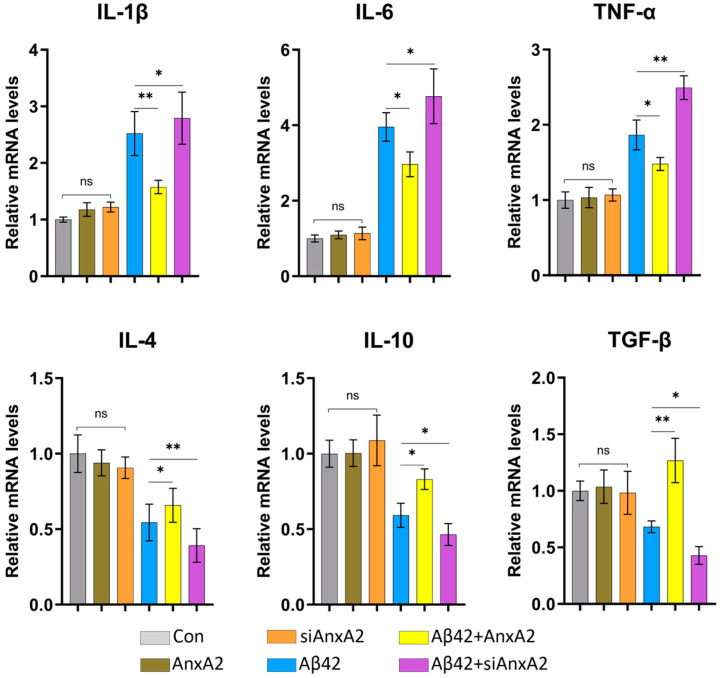
Effects of AnxA2 on mRNA levels of pro- and anti-inflammatory cytokines in SH-SY5Y cells. Proinflammatory cytokines include IL-1β, IL-6, and TNF-α, and anti-inflammatory cytokines include IL-4, IL-10, and TGF-β. Data are presented as mean ± SEM. * *p* < 0.05, ** *p* < 0.01, ns, not significant, determined by one-way ANOVA, followed by the Tukey–Kramer test for multiple comparisons, n = 4.

Inflammation is a normal defense mechanism of the body against infection, toxins, and injury. However, when the balance between anti-inflammatory and pro-inflammatory actions is disrupted, chronic neuroinflammation may occur. We used qRT-PCR to detect the mRNA levels of pro-inflammatory and anti-inflammatory cytokines in cells. The results showed that in the Aβ42 + AnxA2 group, the mRNA levels of pro-inflammatory cytokines, IL-1β, IL-6, and TNF-α were significantly decreased compared to Aβ42 secreting cells, while the mRNA levels of anti-inflammatory cytokines, IL-4, IL-10, and TGF-β were significantly increased (Figure 11). Conversely, in the Aβ42 + siAnxA2 group, the mRNA levels of pro-inflammatory cytokines, IL-1β, IL-6, and TNF-α were significantly increased, and the mRNA levels of anti-inflammatory cytokines, IL-4, IL-10, and TGF-β were significantly decreased compared to Aβ42-secreting cells. There were no significant differences in the mRNA levels of pro-inflammatory cytokines (IL-1β, IL-6, and TNF-α) and anti-inflammatory cytokines (IL-4, IL-10, and TGF-β) among the Con, AnxA2, and siAnxA2 groups (Figure 11). These data indicate that overexpression of AnxA2 in SH-SY5Y cells can alleviate the inflammatory response caused by secreted Aβ42, whereas the absence of the AnxA2 gene exacerbates the pro-inflammatory response and weakens the anti-inflammatory response.

### 3.11. AnxA2 Gene Knockdown Promotes Apoptosis and Inhibits Autophagy

**Figure 12:**
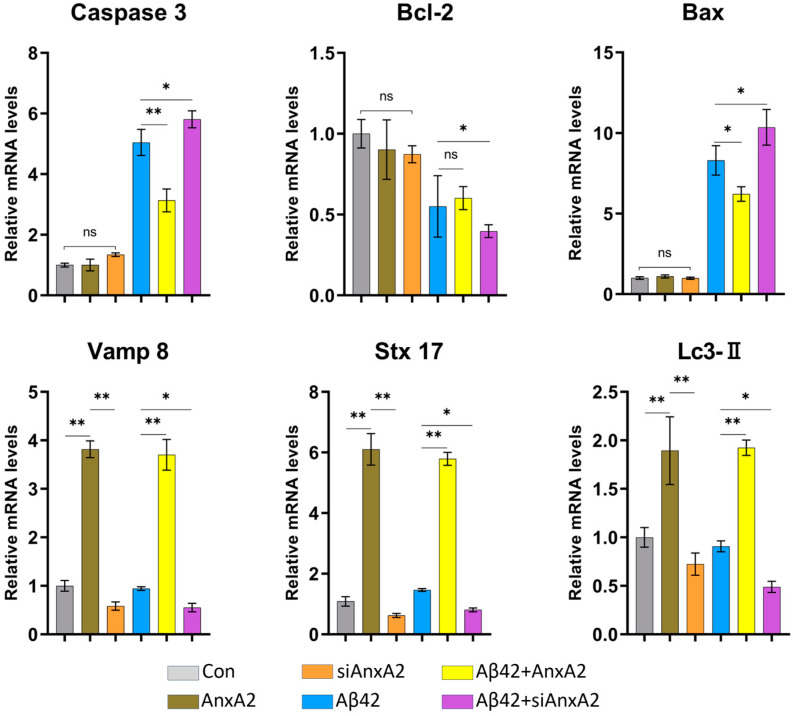
AnxA2 gene knockdown promotes apoptosis and inhibits autophagy in SH-SY5Y cells secreting Aβ42. Data are presented as mean ± SEM. * *p* < 0.05, ** *p* < 0.01, ns, not significant, by one-way ANOVA, followed by the Tukey–Kramer test for multiple comparisons, n = 4.

The transcription levels of key apoptotic and autophagy-related proteins were detected in six groups using qRT-PCR (Figure 12). These proteins include Caspase-3, the main terminal shearing enzyme in apoptosis, Bcl-2, an apoptosis inhibitor, Bax, a pro-apoptotic protein, and the autophagy-related proteins, Vamp8, Stx-17, and Lc3-II. The results showed that in the Aβ42 + AnxA2 group, the mRNA levels of Caspase-3 and Bax were significantly lower (*p* < 0.05) compared to Aβ42-secreting cells, while the mRNA level of Bcl-2 was not significantly different. Conversely, in the Aβ42 + siAnxA2 group, the mRNA levels of Caspase-3 and Bax were significantly higher (*p* < 0.05), and the mRNA levels of Bcl-2 were significantly lower (*p* < 0.05) compared to Aβ42-secreting cells. There were no significant differences in the mRNA levels of Caspase-3, Bcl-2, and Bax among the Con, AnxA2, and siAnxA2 groups. These data indicate that overexpression of AnxA2 in SH-SY5Y cells can alleviate apoptosis induced by secreted Aβ42, while the absence of AnxA2 exacerbates apoptosis.

Furthermore, qRT-PCR results showed that the mRNA levels of Vamp8, Stx-17, and Lc3-II were significantly higher (*p* < 0.01) in the AnxA2-overexpressing group compared to the Con and siAnxA2 groups. The mRNA levels of Vamp8, Stx-17, and Lc3-II in the Aβ42 group were significantly lower (*p* < 0.01) compared to the Aβ42 + AnxA2 group and significantly higher (*p* < 0.05) compared to the Aβ42 + siAnxA2 group. These data suggest that AnxA2 plays a crucial role in autophagy, where Aβ42 secreted by SH-SY5Y cells inhibits autophagy. Overexpression of AnxA2 can mitigate this inhibition, whereas the absence of AnxA2 exacerbates the inhibition of autophagy.

## 4. Discussion

Based on the analysis results from the GEO and HG databases, the expression levels of hub genes were further identified by qRT-PCR in AD cell models (SH-SY5Y and HMC3). The results of qRT-PCR showed that the expression levels of MAPK8, NOTCH1, TLR4, AnxA2, APP, CREB1, HIF1A, ITGB1, and KRAS were consistent with the clinical bioinformatic data. Notably, many of these genes have been well-studied in Alzheimer’s disease, while others, such as the AnxA2 gene, a hub gene recently reported to be associated with AD in the hippocampus [53], remain poorly understood regarding their role in AD. To investigate the molecular mechanisms by which AnxA2 affects AD pathophysiology, transcriptome sequencing was used to explore the impact of AnxA2 gene knockdown on transcriptional levels in an AD cell model that secretes Aβ42. Transcriptome sequencing analysis revealed that AnxA2 knockdown primarily affects oxidative phosphorylation, cell cycle, AD-related genes, protein processing in the endoplasmic reticulum, SNARE interactions in vesicle transport, and autophagy. The changes in gene expression suggest that AnxA2 is involved in multiple critical cellular functions and signaling pathways [54]. Specifically, the downregulation of oxidative phosphorylation-related gene DN5 indicates that AnxA2 deletion may impact mitochondrial function, thereby affecting neuronal energy metabolism [55]. The downregulation of cell cycle-related genes TGF-β, Ink4c, and CDK4/6 suggests that AnxA2 may play an important role in cell cycle regulation [56]. Additionally, the upregulation of AD-related genes PSEN and ApoE leads to the production of more Aβ42, increased Aβ deposition, tau hyperphosphorylation, and accelerated cognitive decline [57], further supporting the significance of AnxA2 in AD pathogenesis. The downregulation of genes related to protein processing in the endoplasmic reticulum and vesicle transport suggests that AnxA2 may play a role in cellular stress response and homeostasis by affecting protein folding and transport processes [58]. The downregulation of autophagy-related genes, Vamp8, Stx-17, and Snap29, indicates that AnxA2 might play a crucial regulatory role in the autophagy pathway, which is vital for maintaining cellular homeostasis and responding to cellular damage [59].

Flow cytometry analysis showed that AnxA2 knockdown significantly increased Aβ42-induced neuronal apoptosis rates (Figure 6 and Figure S7). Changes in mRNA levels of Caspase-3, Bcl-2, and Bax (Figure 12) indicate that AnxA2 knockdown exacerbates apoptosis via the intrinsic pathway. This aligns with AnxA2’s role in inhibiting apoptosis in other cell types by regulating the cytoskeleton and signaling pathways [60]. Apoptosis is a key feature of AD pathophysiology, and Aβ42 can induce neuronal apoptosis through oxidative stress and inflammatory responses [13]. AnxA2 knockdown leads to upregulation of Caspase-3 and downregulation of Bcl-2, further promoting Aβ42-induced apoptosis [61]. Increased ROS levels are a significant aspect of AD pathophysiology. Our results showed that AnxA2 knockdown significantly raised Aβ42-induced ROS levels (Figure 7 and Figure S8), suggesting that AnxA2 might influence cellular antioxidant capacity by regulating the Nrf2/ARE-signaling pathway [62]. Nrf2 is a key transcription factor that regulates the expression of various antioxidant enzymes and is controlled by AnxA2 [63]. In AD pathology, Aβ42 promotes oxidative stress by inducing mitochondrial damage and ROS production [55]. AnxA2 knockdown resulted in a significant increase in ROS levels, potentially exacerbating cell damage by affecting Nrf2 and other antioxidant pathways [64].

Analysis of cell cycle distribution by PI staining and flow cytometry revealed that AnxA2 knockdown caused significant accumulation of cells in the G2 phase and a decrease in the proportion of S phase cells (Figure 8). This suggests that AnxA2 may play a crucial role in the proliferation of cells, possibly by regulating the expression of Cyclin D1 and CDK4 [56]. Cell cycle regulation is essential for cell proliferation and differentiation. AnxA2 knockdown led to G1 phase accumulation, potentially impeding S phase entry by affecting genes such as TGF-β and Ink4c [65]. This cell cycle disruption may exacerbate neuronal aging and apoptosis [66]. ATP is a critical molecule for cellular energy metabolism. ATP fluorescent assays showed that AnxA2 knockdown significantly reduced ATP levels in the AD cell model (Figure 9), indicating that AnxA2 may regulate energy metabolism through the modulation of mitochondrial function or glucose metabolism pathways [67]. Further experiments demonstrated that AnxA2 deficiency led to decreased mitochondrial membrane potential and increased mitochondrial oxidative stress, underlying its essential role in maintaining mitochondrial function [68]. Mitochondrial dysfunction is a critical component of AD pathophysiology, as Aβ42 can damage mitochondria through various mechanisms, leading to reduced ATP production and increased oxidative stress [69,70]. AnxA2 knockdown exacerbated mitochondrial damage, highlighting its importance in mitochondrial function and energy metabolism.

Changes in cell migration ability may influence the progression of AD pathology. By wound healing and transwell migration assays, we found that AnxA2 knockdown significantly inhibited Aβ42-induced neuronal cell migration (Figure 10 and Figure S9). This is likely due to AnxA2’s role in cytoskeletal reorganization and focal adhesion dynamics, affecting cell migration and invasion abilities. Cell migration is a crucial physiological process in multicellular organisms, playing key roles in neural development and injury repair [71]. AnxA2 promotes cell migration by regulating the cytoskeleton and cell adhesion molecules [72]. AnxA2 knockdown leading to reduced migration capability may further impact neuronal function and survival [65].

qRT-PCR analysis showed that after AnxA2 knockdown, the mRNA levels of pro-inflammatory cytokines (IL-1β, IL-6, and TNF-α) were significantly upregulated, while anti-inflammatory cytokines (IL-4, IL-10, and TGF-β) decreased significantly (Figure 11). This suggests that AnxA2 may exert an anti-inflammatory effect in the AD cell model by negatively regulating the proinflammatory cytokines. Inflammatory responses are also a crucial aspect of AD pathology, with Aβ42 capable of triggering inflammation through the activation of microglia and astrocytes [73,74]. AnxA2 knockdown leading to upregulation of proinflammatory and downregulation of anti-inflammatory cytokines indicates its potential role in modulating inflammation in AD. Apoptosis-related genes, such as Caspase-3, Bcl-2, and Bax, showed significant changes in mRNA levels after AnxA2 knockdown (Figure 12). The increased Bax/Bcl-2 ratio supports the previously observed increase in apoptosis rates, further demonstrating AnxA2’s role in apoptosis regulation. Apoptosis is a key process in AD pathophysiology, with Aβ42 inducing neuronal apoptosis through various pathways [75,76]. AnxA2 knockdown led to Bcl-2 downregulation and Bax upregulation, further promoting the Caspase-3-mediated apoptotic pathway. Autophagy-related genes, such as Vamp8, Stx-17, and Lc3-II, showed significant downregulation in mRNA transcription after AnxA2 knockdown (Figure 12). This suggests that AnxA2 may play a crucial role in AD pathophysiology by regulating the autophagy pathway. Autophagy is an essential intracellular degradation pathway critical for maintaining cellular homeostasis and responding to cellular damage [77,78]. AnxA2 knockdown leading to the downregulation of autophagy-related genes may impair autophagosome formation and function, thereby exacerbating Aβ42-induced cellular damage. Because of the complexities of the experimental findings, more in vitro and in vivo investigations are expected to clarify the pathophysiological role of AnxA2 in AD.

## 5. Conclusions

In the present study, a total of 85 DEGs and 20 hub genes were identified. Through systematic analysis of the multifaceted effects of AnxA2 gene knockdown on the cell model of AD, we revealed the role of AnxA2 in regulating apoptosis, ROS levels, cell cycle, energy metabolism, cell migration, and inflammatory responses. In the AD cell model, AnxA2 gene silencing exacerbated Aβ42-induced neuronal damage, apoptosis, elevated ROS levels, and cell cycle abnormalities while reducing ATP levels, indicating its essential role in mitochondrial function. Additionally, AnxA2 deficiency intensified pro-inflammatory responses and inhibited autophagy and cell migration, whereas AnxA2 overexpression attenuated Aβ42-induced inflammatory response. These findings suggest that AnxA2 may play a protective role in the pathophysiology of AD. This study may provide a theoretical basis for the development of new medicines to combat neurodegenerative diseases in the future.

## Data Availability

The datasets generated and/or analyzed during the current study are available in the following public repositories: Gene expression profiles (GSE5281 and GSE97760) were obtained from the Gene Expression Omnibus (GEO) database (https://www.ncbi.nlm.nih.gov/geo/, accessed on 26 August 2022). The gene-related information used in this study was retrieved from GeneCard: The Human Gene Database (https://www.genecards.org/, accessed on 26 August 2022). All relevant datasets are publicly accessible.

## Supplementary Materials

The following supporting information can be downloaded at: https://www.mdpi.com/article/10.3390/antiox13101274/s1, Figure S1. Enrichment analysis of the microarray data. Figure S2. PPI network and the most significant module of differentially expressed genes (DEGs) between AD-related and membrane protein-related genes. Figure S3. Spearman correlation analysis of the 85 differentially expressed genes (DEGs). Figure S4. The landscape of immune infiltration between Alzheimer’s disease (AD) and normal controls. Figure S5. Transcriptional abundance of MAPK8, NOTCH1, TLR4, AnxA2, APP, CREB1, HIF1A, ITGB1 and KRAS in Alzheimer’s disease (AD) patients in comparison to the control group, measured by microarray or RNA-seq. Figure S6. RNA levels of hub genes measured in Aβ-secreting and non-secreting cells. Figure S7. Flow cytometry analysis of the effects of siAnxA2/P11 on cell apoptosis. Figure S8. Effects of siAnxA2/P11 on ROS levels in Aβ42-secreting HMC3 cells. Figure S9. Role of AnxA2 in cell migration.
