# Supplementary material for: Integrative Human Genetic and Cellular Analysis of the Pathophysiological Roles of AnxA2 in Alzheimer’s Disease
Source: Antioxidants (Basel). 2024 Oct 21;13(10):1274. doi: 10.3390/antiox13101274 (PMC11504888; doi:10.3390/antiox13101274)
Supplement: Supplementary file 1 [file antioxidants-13-01274-s001.zip › antioxidants-3213523-supplementary.pdf]

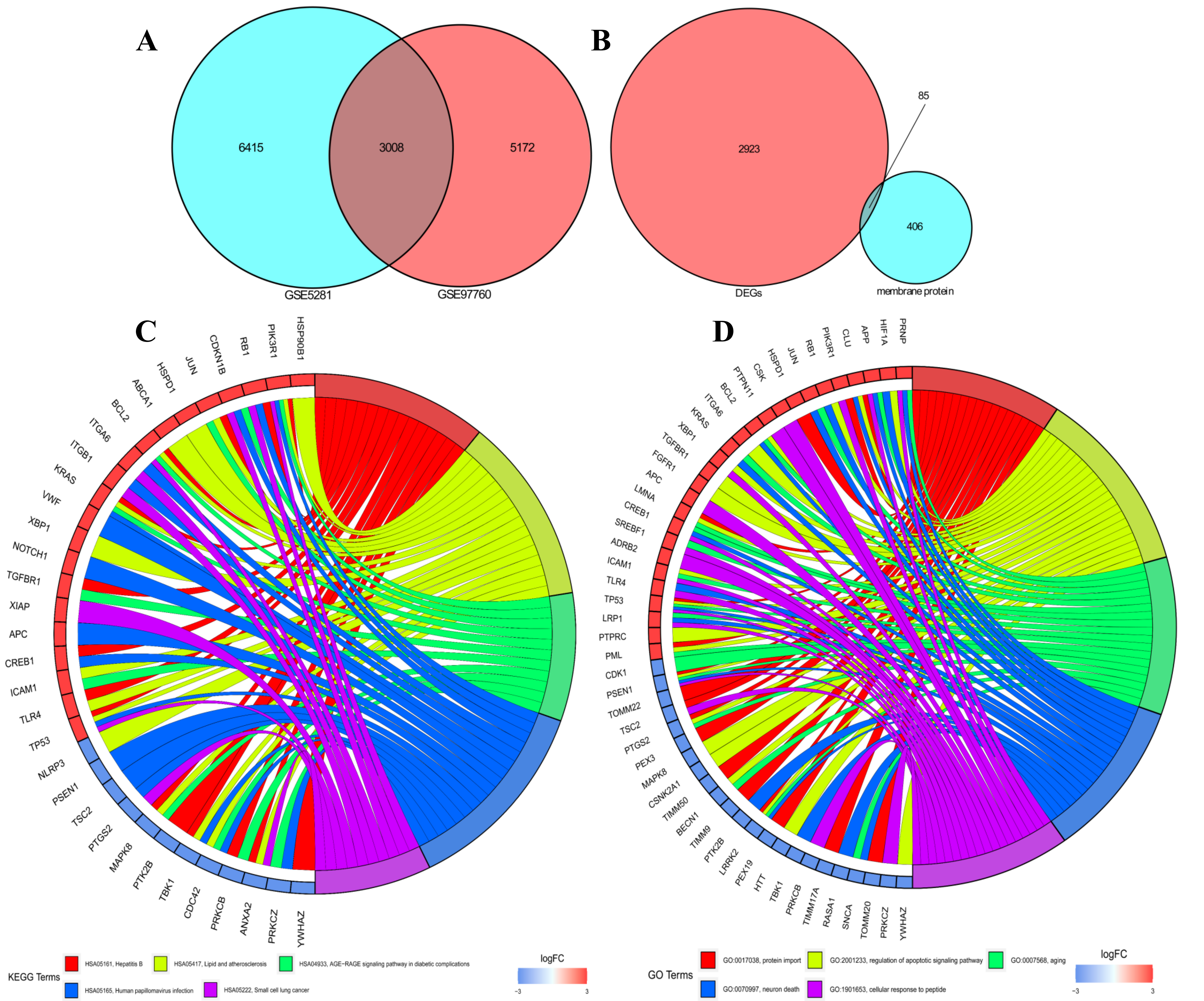

Figure S1. Enrichment analysis of the microarray data. (A) Venn diagram showing the numbers of overlapped DEGs genes between the GSE5281 dataset and the GSE97760 dataset. (B) The Venn diagram shows the overlapped genes (OLGs) between AD-related modules genes and membrane protein-related genes. (C) Gene Ontology (GO) functional analysis showing enrichment of target genes. (D) Kyoto Encyclopedia of Genes and Genomes (KEGG) pathway enrichment analysis of target genes.

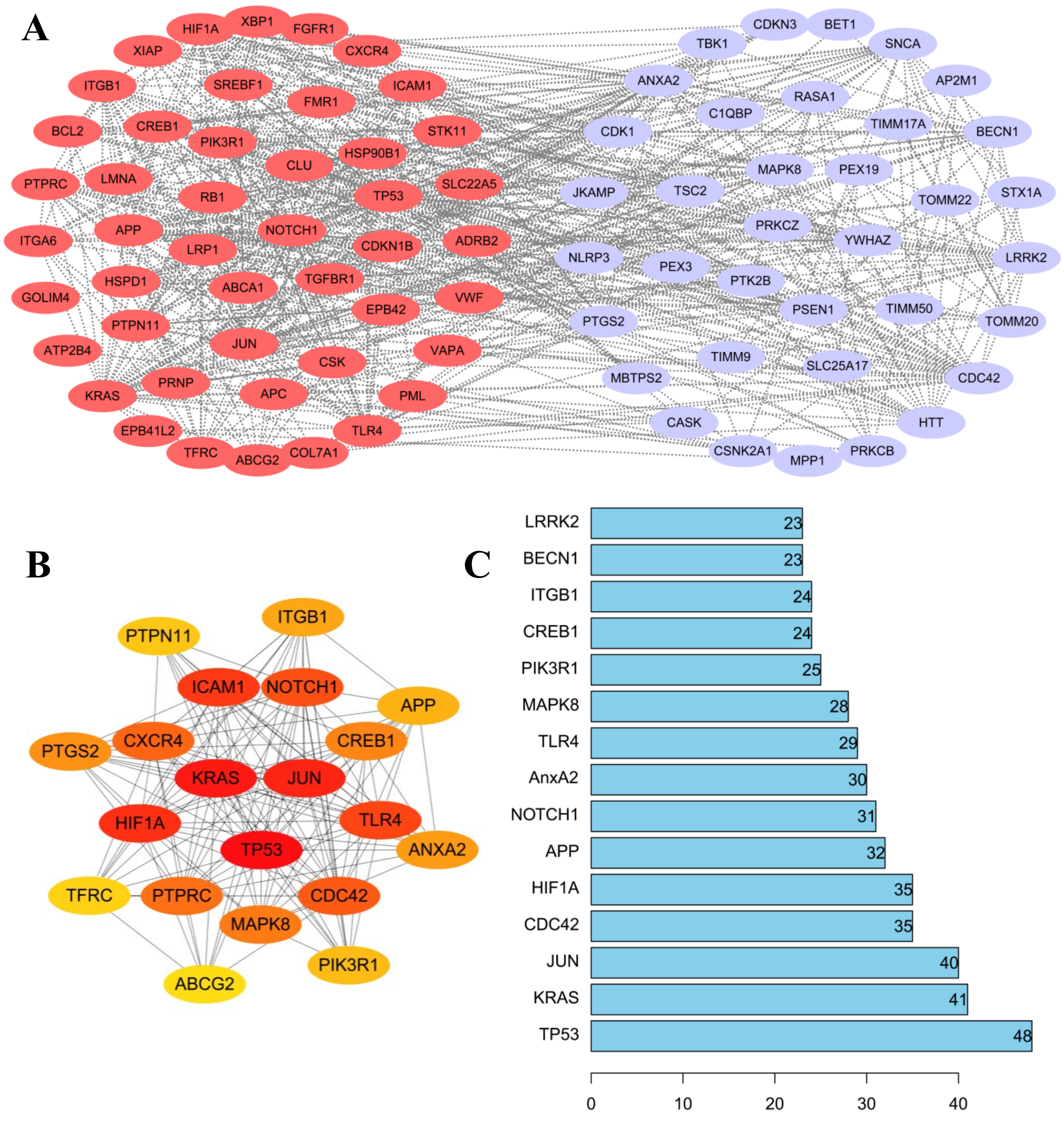

Figure S2. PPI network and the most significant module of differentially expressed genes (DEGs) between AD-related and membrane protein-related genes. (A) The PPI network of DEGs was constructed using Cytoscape. (B) The most significant module was obtained from the PPI network with 20 hub genes. Upregulated genes are marked in red; downregulated genes are marked in blue. (C) The interaction number of each differentially expressed hub gene.

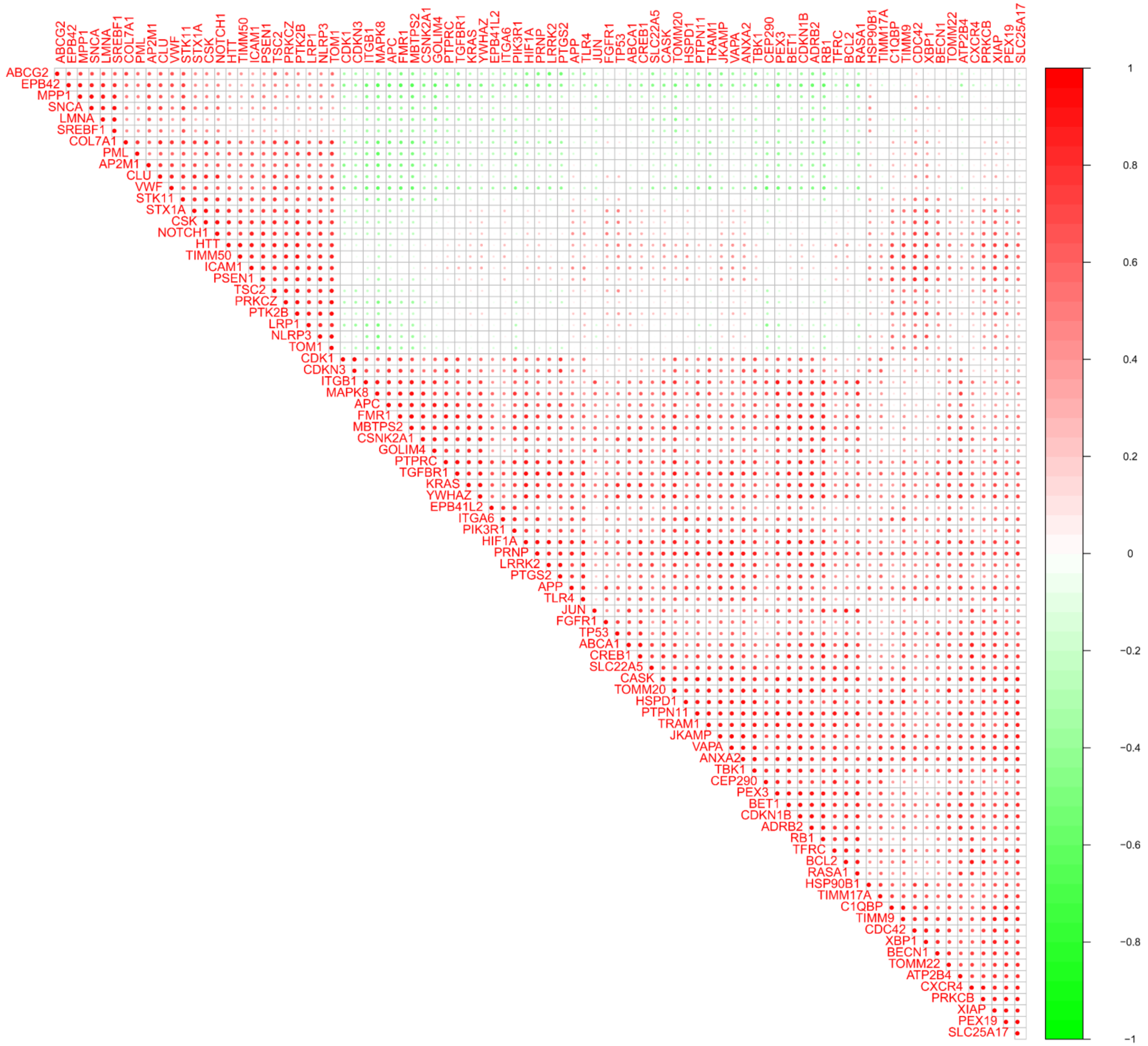

Figure S3. Spearman correlation analysis of the 85 differentially expressed genes (DEGs) .

A

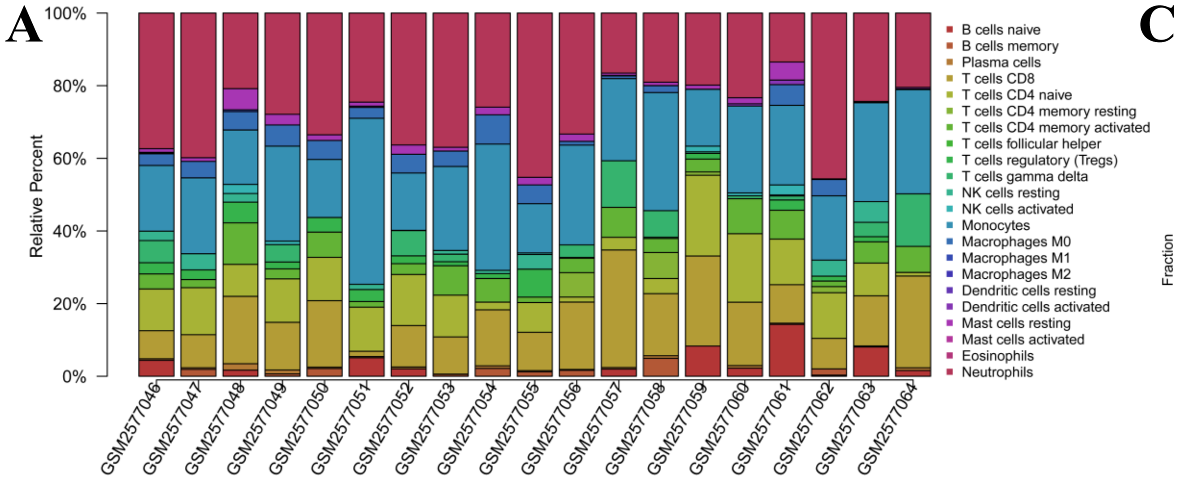

C

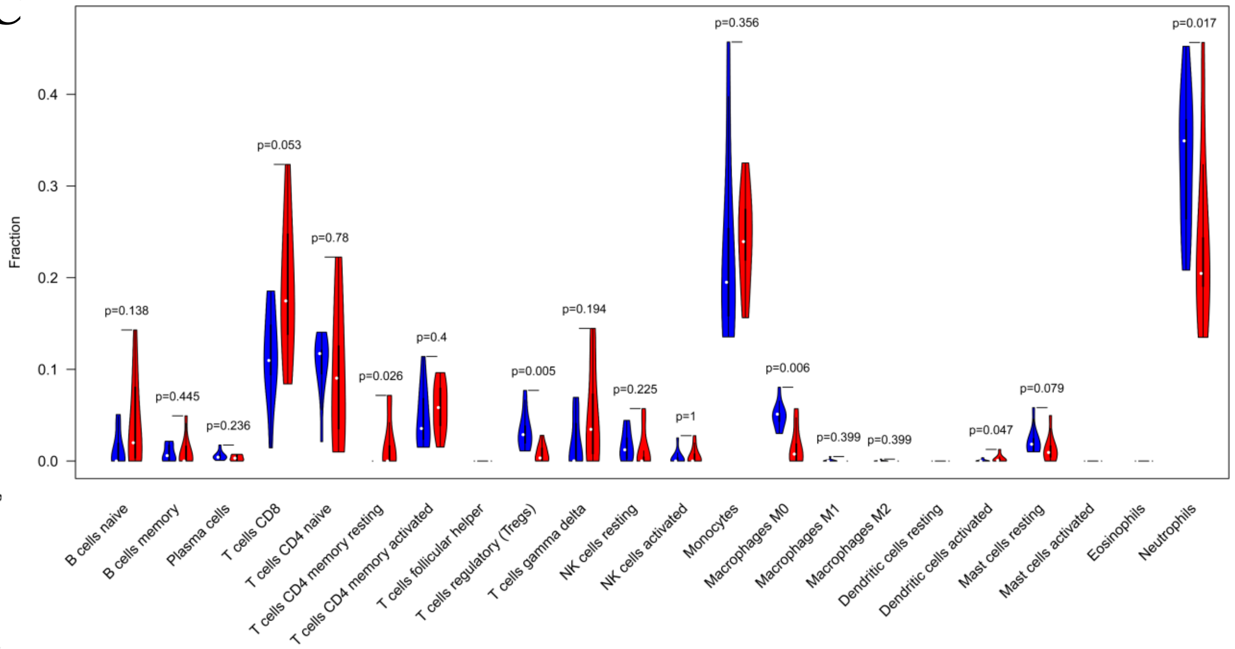

B

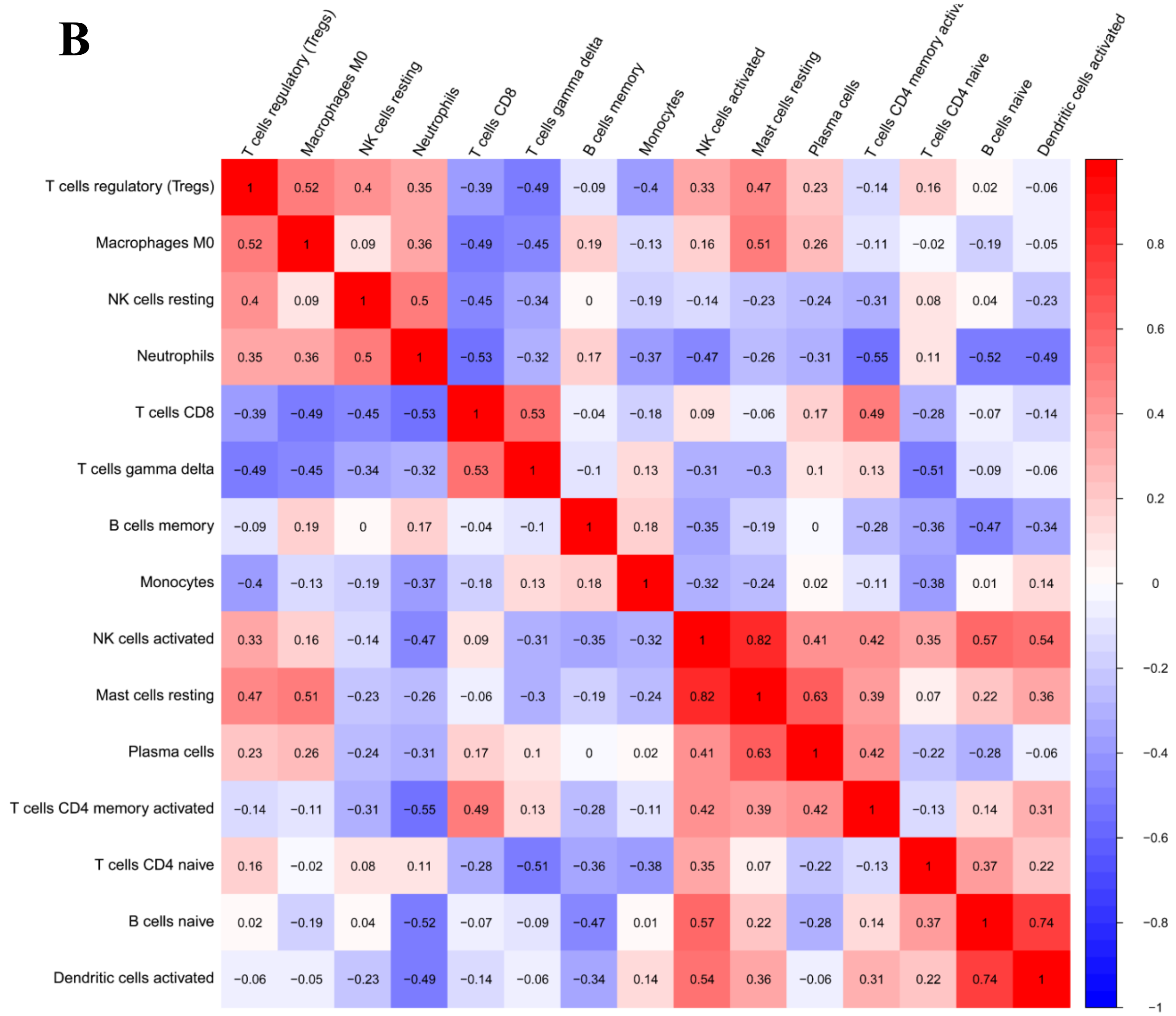

Figure S4. The landscape of immune infiltration between Alzheimer's disease (AD) and normal controls. (A) The relative percentage of 22 subpopulations of immune cells in 20 samples from GSE5281 and GSE97760 datasets. (B) Spearman correlation analysis of the 22 subpopulations of immune cells in datasets. (C) The difference in immune infiltration between AD and normal controls. The normal controls group was marked as blue and the AD group as red. An FDR-adjusted P value less than 0.05 was considered as statistical significance.

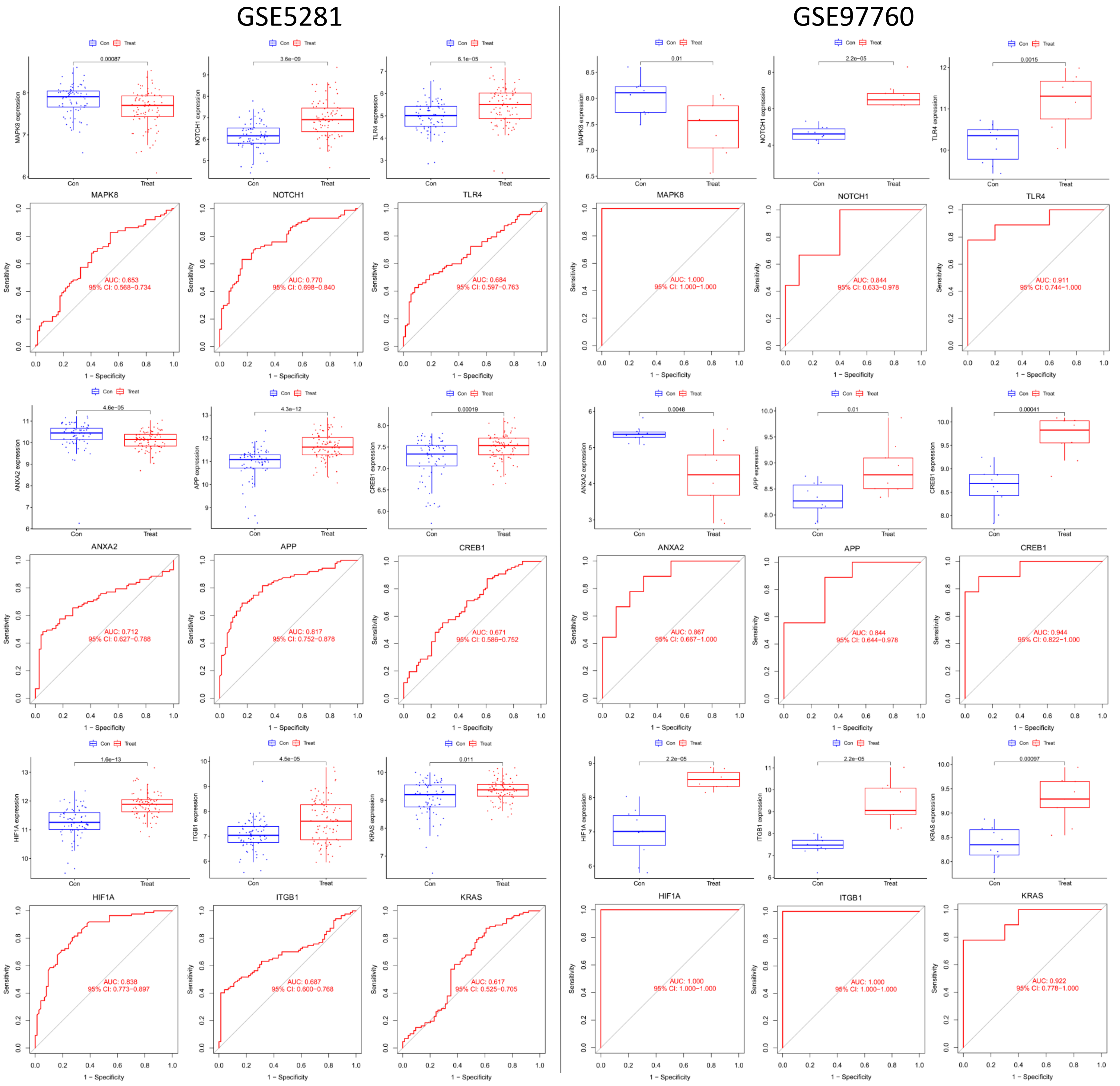

Figure S5. Transcriptional abundance of MAPK8, NOTCH1, TLR4, AnxA2, APP, CREB1, HIF1A, ITGB1 and KRAS in Alzheimer's disease (AD) patients in comparison to the control group, measured by mi-croarray or RNA-seq.The ROC curve for each dataset was located below the corresponding dot plot. The area under the curves (AUC) for all ROC curves was used to predict diagnostic value.

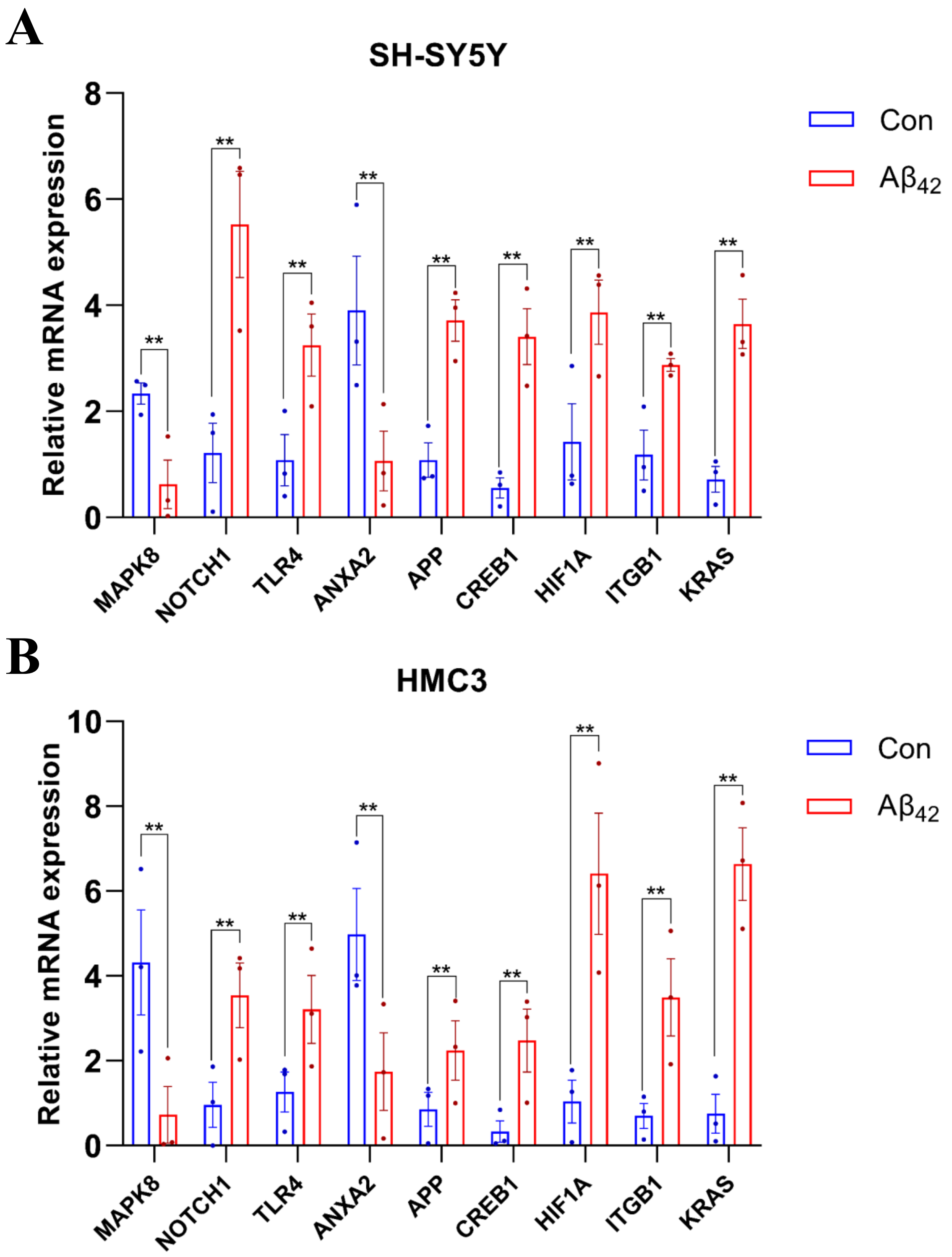

Figure S6. RNA levels of hub genes were measured in Aβ-secreting and non-secreting cells. RNA expression of MAPK8, NOTCH1, TLR4, AnxA2, APP, CREB1, HIF1A, ITGB1 and KRAS were measured in SH-SY5Y cell (A) and HMC3 cell (B) samples using qRT-PCR. All experiments were performed in triplicate, and results were presented as Mean with SEM. ** $p<0.01$, analyzed by one-way ANOVA, followed by the Tukey-Kramer test for multiple comparisons, n = 6..

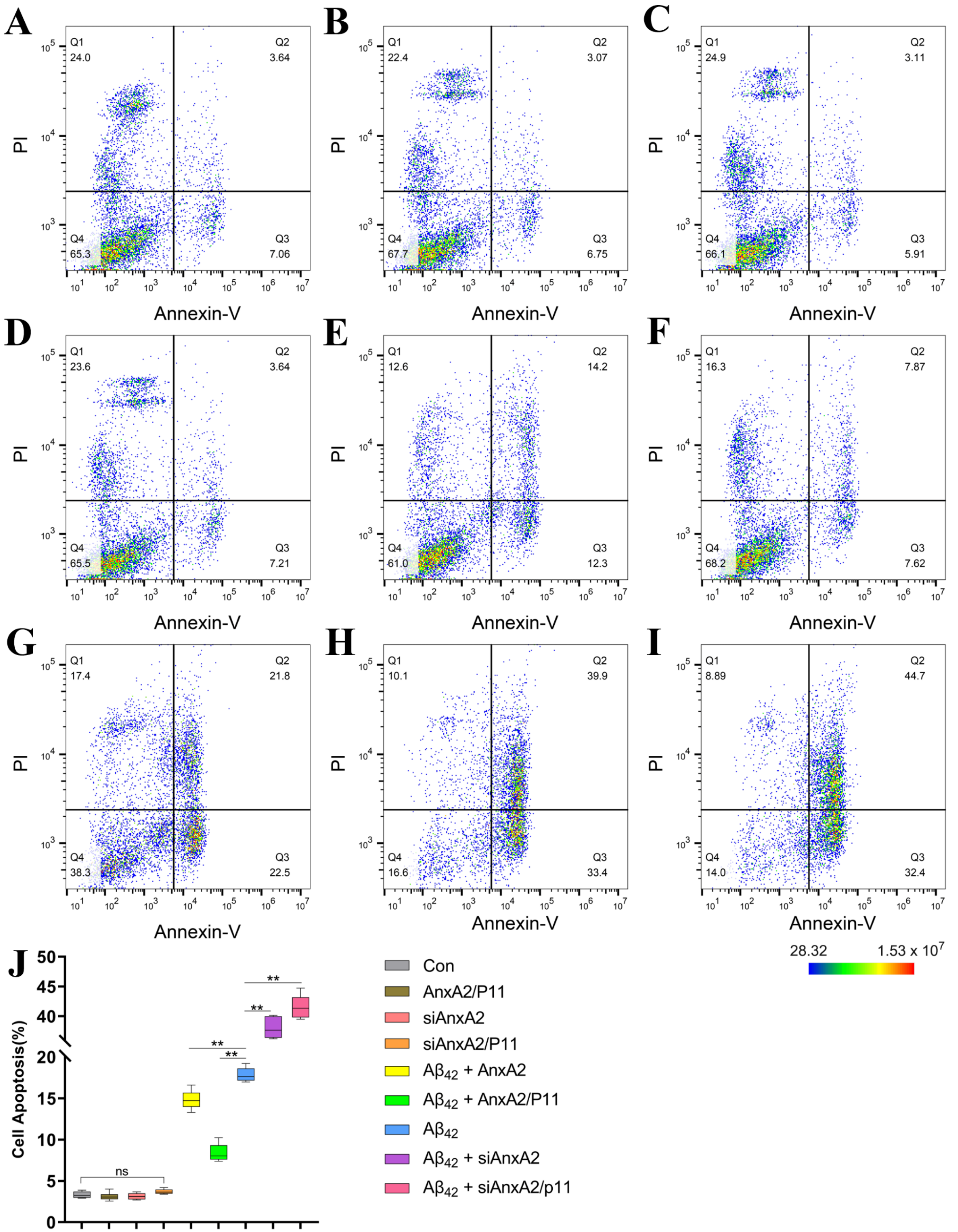

Figure S7. Flow cytometry analysis of the effects of siAnxA2/P11 on cell apoptosis. (A) Apoptosis in HMC3 cells treated with control medium (Con). (B) Apoptosis in HMC3 cells overexpressing AnxA2/P11. (C) Apoptosis in siAnxA2-treated HMC3 cells. (D) Apoptosis in siAnxA2/P11-treated HMC3 cells. (E) Apoptosis in Aβ42-secreting HMC3 cells overexpressing AnxA2. (F) Apoptosis in Aβ42-secreting HMC3 cells overexpressing AnxA2/P11. (G) Apoptosis in HMC3 cells secreting Aβ42. (H) Apoptosis in Aβ42-secreting HMC3 cells treated with siAnxA2. (I) Apoptosis in Aβ42-secreting HMC3 cells treated with siAnxA2/P11. (J) Quantification of apoptosis in the HMC3 cells using FlowJoTM. Results are expressed as mean ± SEM. $**p<0.01$, analyzed by one-way ANOVA, followed by the Tukey-Kramer test for multiple comparisons, n = 6.

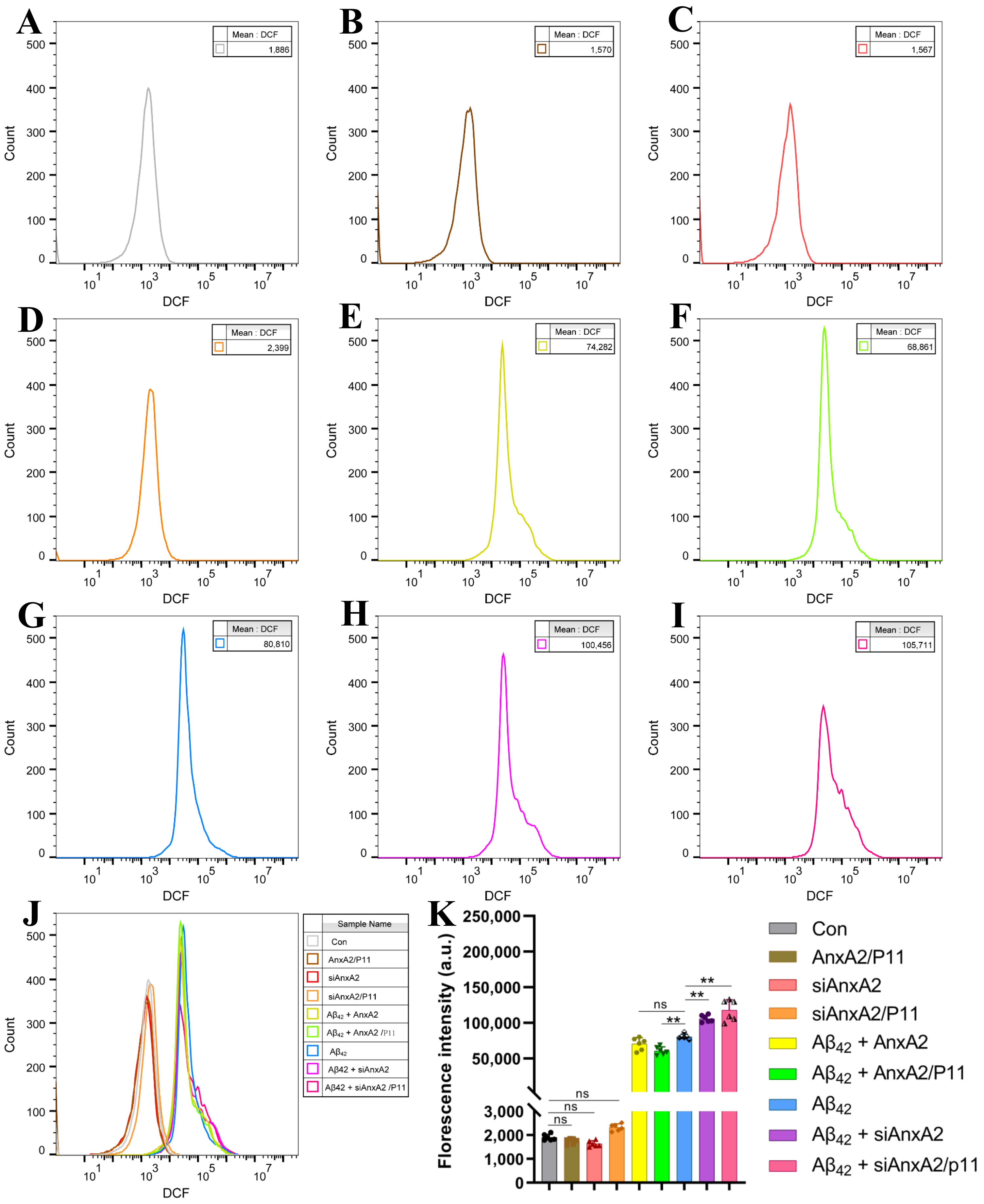

Figure S8. Effects of siAnxA2/P11 on ROS levels in Aβ42–secreting HMC3 cells. (A) ROS in wild type HMC3 cells (control). (B) ROS produced in HMC3 cells overexpressing AnxA2/P11. (C) ROS levels in siAnxA2-treated HMC3 cells. (D) ROS levels in siAnxA2/P11-treated HMC3 cells. (E) ROS levels in Aβ42-secreting HMC3 cells overexpressing AnxA2. (F) ROS levels in Aβ42-secreting HMC3 cells overexpressing AnxA2/P11. (G) ROS levels in Aβ42-secreting HMC3 cells. (H) ROS levels in Aβ42-secreting HMC3 cells treated with siAnxA2. (I) ROS levels in Aβ42-secreting HMC3 cells treated with siAnxA2/P11. (J) Overlay of flow cytometry charts shown in figure A to I. (K) Quantification of the ROS levels using FlowJoTM. Results are expressed as mean ± SEM. $^{**}p<0.01$, analyzed by one-way ANOVA, followed by the Tukey-Kramer test for multiple comparisons, n = 6.

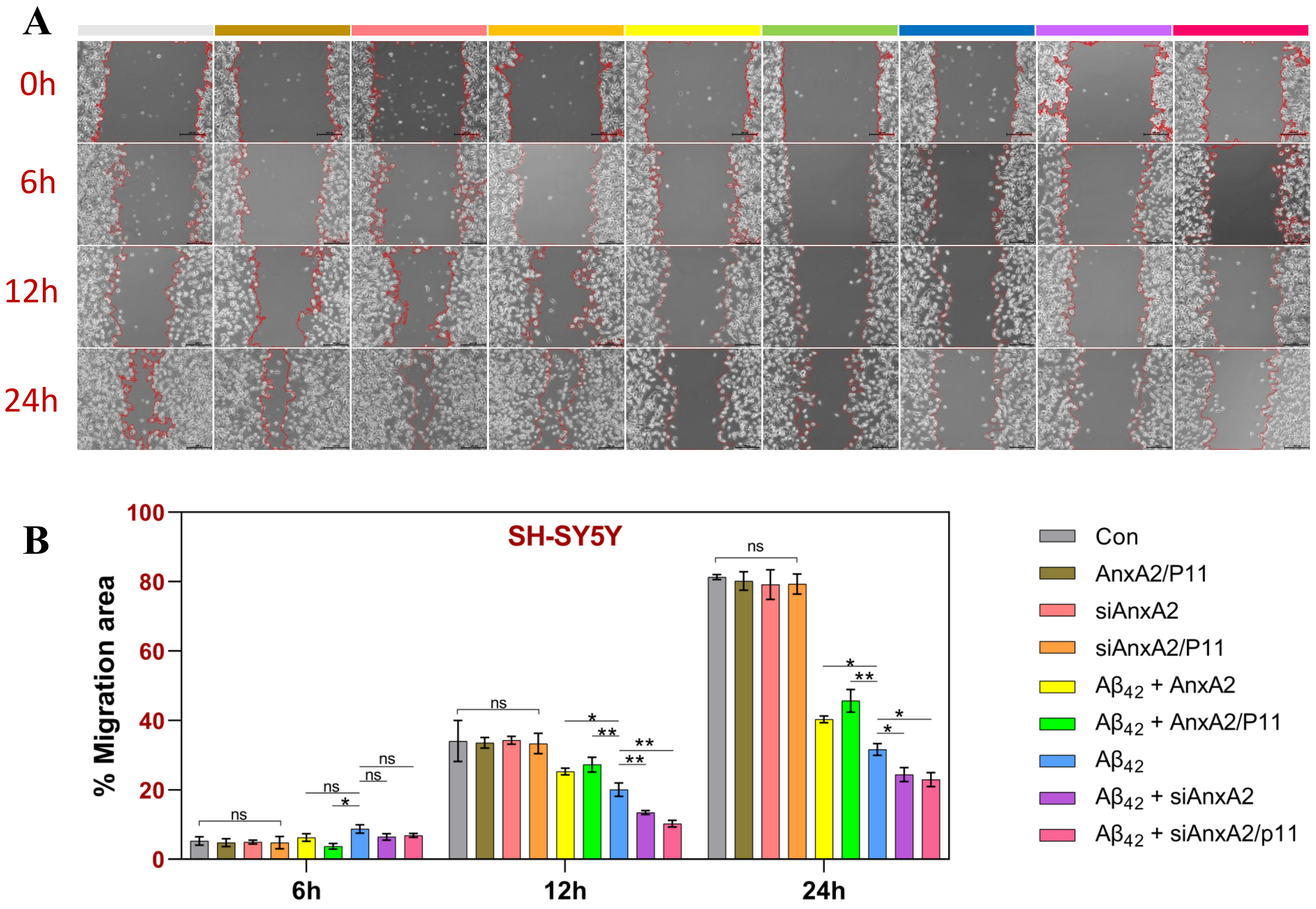

Figure S9. Role of AnxA2 in cell migration. (A) Microscopic images of cells at 0, 6, 12, and 24 h after scratching; (B) Statistical analysis of cell migration using the ImageJ software. Data are presented as mean ± SEM. $*p<0.05$, $**p<0.01$, analyzed by one-way ANOVA, followed by Tukey-Kramer test for multiple comparisons, n = 6.
